# Supplementary material for: Estimating population immunity against serotype-two poliomyelitis from the inactivated polio vaccine in routine immunization across 112 countries: A modelling study
Source: PLoS Med. 2026 Mar 19;23(3):e1004952. doi: 10.1371/journal.pmed.1004952 (PMC13001910; doi:10.1371/journal.pmed.1004952)
Supplement: S1 Text — (PDF) [file pmed.1004952.s001.pdf]

Supplementary Material

Estimating population immunity against type-2 poliomyelitis from the inactivated polio vaccine in routine immunisation across 112 countries: a modelling study

Elizabeth J Gray, Alejandro Ramirez Gonzalez, Ondrej Mach, Nieves Derqui, Nicholas Grassly, Laura V Cooper, Isobel M Blake

Contents

1 Country schedules and modelled routine immunisation coverages by region 3

1.1 African Region Countries . . . 3

1.2 Americas Region Countries . . . 4

1.3 Eastern Mediterranean Region Countries . . . 4

1.4 European Region Countries . . . 5

1.5 South East Asia Region Countries . . . 5

1.6 Western Pacific Region Countries . . . 5

2 Immunity projections 6

2.1 African Region countries . . . 6

2.2 Americas Region countries . . . 12

2.3 Eastern Mediterranean Region Countries . . . 16

2.4 European Region Countries . . . 18

2.5 South East Asia Region Countries . . . 19

2.6 Western Pacific Region Countries . . . 21

3 Gains from switching to 14, 39 week schedule 23

3.1 African Region countries . . . 23

3.1.1 Single dose countries . . . 23

3.1.2 Two-dose countries . . . 24

3.2 Americas Region Countries . . . 24

3.2.1 Single dose countries . . . 24

3.2.2 Two-dose countries . . . 24

3.3 Eastern Mediterranean countries . . . 25

3.3.1 Two-dose countries . . . 25
3.4 European Region Countries . . . 25
3.4.1 Two-dose countries . . . 25
3.5 South East Asia Region countries . . . 26
3.5.1 One dose countries . . . 26
3.5.2 Two-dose countries . . . 26
3.6 Western Pacific Region Countries . . . 26
3.6.1 Single dose countries . . . 26
3.6.2 Two-dose countries . . . 27
3.7 Equilibrium immunity estimates: accounting for delay . . . 28

4 Methods **29**
4.1 Coverage decline model . . . 29
4.2 Seroconversion with age model . . . 29
4.2.1 Full dose model . . . 30
4.2.2 Fractional dose model . . . 31
4.3 Immunity estimates 2024-2031 . . . 32
4.4 Estimates with catch-ups . . . 32
4.5 Equilibrium immunity with delay. . . . 33

5 Additional Figures **34**
5.1 Delay in vaccine administration . . . 34
5.2 Coverage decline (WUENIC 2023) in countries considered . . . 34

6 Number of countries administering different IPV dose numbers per year **37**

1 Country schedules and modelled routine immunisation coverages by region

1.1 African Region Countries

Table A: Country schedules and modelled routine immunisation coverages: African region countries.

Country	Age at first dose (weeks)	Age at second dose (weeks)	Modelled Coverage			Second dose introduced
			6 weeks	14 weeks	39 weeks	
Angola	17	39	62%	57%	41%	2022
Benin	14		81%	75%	48%	
Botswana	17	39	92%	91%	89%	2024
Burkina Faso	16	39	95%	94%	90%	2021
Burundi	14		89%	88%	85%	
Cabo Verde	17	39	96%	95%	92%	2023
Cameroon	14	39	82%	79%	63%	2023
Central African Republic	14		51%	48%	38%	
Chad	14	39	82%	77%	53%	2021
Comoros	14	39	79%	78%	77%	2025
Congo	16	36	75%	71%	55%	2025
Cote d'Ivoire	6	14	80%	77%	65%	2023
Democratic Republic of the Congo	14	39	71%	64%	39%	2023
Equatorial Guinea	14		81%	77%	59%	
Eritrea	14	39	96%	96%	93%	2021
Eswatini	14	39	77%	77%	77%	2024
Ethiopia	14	39	73%	71%	62%	2024
Gabon	14		75%	72%	62%	
Gambia	17	39	77%	77%	77%	2022
Ghana	14	39	96%	95%	89%	2024
Guinea	14		60%	56%	42%	
Guinea-Bissau	14		80%	76%	62%	
Kenya	14		91%	90%	86%	
Lesotho	14	39	84%	84%	84%	2024
Liberia	14		90%	88%	78%	
Madagascar	14	39	69%	66%	53%	2021
Malawi	14	39	92%	91%	83%	2024
Mali	14	39	75%	73%	64%	2021
Mauritania	14		94%	93%	87%	
Mozambique	17	39	85%	81%	65%	2023
Namibia	14	39	85%	83%	77%	2023
Niger	14	39	90%	88%	80%	2022
Nigeria	6	14	61%	59%	52%	2021
Rwanda	14	39	96%	95%	93%	2022
Sao Tome and Principe	14	39	80%	80%	80%	2024
Senegal	6	14	86%	84%	75%	2022
Sierra Leone	14	39	90%	89%	84%	2021
South Sudan	14	39	73%	69%	54%	2021
Togo	14	39	88%	86%	72%	2022

Uganda	6	14	92%	89%	72%	2022
United Republic of Tanzania	14	39	94%	93%	89%	2025
Zambia	14	39	74%	74%	74%	2024
Zimbabwe	14	39	90%	89%	86%	2021

1.2 Americas Region Countries

Table B: Country schedules and modelled routine immunisation coverages: Americas region countries.

Country	Age at first dose (weeks)	Age at second dose (weeks)	Modelled Coverage			Second dose introduced
			6 weeks	14 weeks	39 weeks	
Anguilla	9	17	97%	96%	90%	2019
Antigua and Barbuda	9	17	95%	95%	95%	2015
Bahamas	9	17	87%	85%	77%	2016
Barbados	9	17	91%	90%	86%	2019
Belize	9	17	86%	86%	86%	2020
Bolivia (Plurinational State of)	9	26	73%	71%	65%	2021
British Virgin Islands	9	26	86%	84%	77%	2019
Cuba	17	34	99%	99%	99%	2018
Curacao	9		NA%	NA%	NA%	
Dominica	9	17	86%	86%	86%	2021
Ecuador	9	17	72%	71%	70%	2018
Grenada	6	26	92%	91%	88%	2021
Guatemala	9	17	86%	86%	85%	2020
Guyana	9	17	96%	96%	96%	2020
Haiti	6		66%	62%	51%	
Honduras	9	17	73%	73%	73%	2018
Jamaica	6	26	98%	98%	95%	2021
Montserrat	9	17	89%	89%	89%	2018
Nicaragua	9	17	88%	88%	88%	2022
Saint Kitts and Nevis	9	26	97%	97%	96%	2023
Saint Lucia	9	26	92%	91%	88%	2023
Saint Vincent and The Grenadines	9	17	97%	96%	94%	2018
Suriname	9	17	80%	79%	72%	2023
Trinidad and Tobago	9	17	98%	98%	96%	2020
Turks and Caicos	9	17	100%	100%	100%	2019
Venezuela	9	17	58%	57%	55%	2021

1.3 Eastern Mediterranean Region Countries

Table C: Country schedules and modelled routine immunisation coverages: Eastern Mediterranean region countries.

Country	Age at first dose (weeks)	Age at second dose (weeks)	Modelled Coverage			Second dose introduced
			6 weeks	14 weeks	39 weeks	
Afghanistan	14	39	62%	59%	52%	2020
Djibouti	6	14	71%	71%	70%	2024
Iran (Islamic Republic of)	17	26	98%	98%	98%	2021
Iraq	17	26	76%	76%	76%	2016
Lebanon	9	17	77%	75%	67%	2023

Morocco	16	39	92%	92%	92%	2025
Oman	9	17	99%	99%	99%	2016
Pakistan	14	39	92%	90%	84%	2021
Occupied Palestinian Territories	4	9	87%	87%	87%	2018
Qatar	9	17	98%	98%	98%	2016
Somalia	14	39	46%	41%	29%	2022
Sudan	6	14	51%	50%	46%	2023
Syrian Arab Republic	8	16	72%	71%	69%	2008
Yemen	14	39	49%	48%	44%	2021

1.4 European Region Countries

Table D: Country schedules and modelled routine immunisation coverages: European region countries.

Country	Age at first dose	Age at second dose	Modelled Coverage			Second dose introduced
	(weeks)	(weeks)	6 weeks	14 weeks	39 weeks	
Albania	9	17	97%	97%	97%	2014
Azerbaijan	13	26	90%	90%	90%	2022
Kyrgyzstan	15	39	89%	89%	89%	2022
Republic of Moldova	26	104	88%	88%	88%	2022
Tajikistan	13	39	99%	99%	98%	2022
Ukraine	9	17	82%	82%	82%	2004
Uzbekistan	17	39	97%	97%	97%	2022

1.5 South East Asia Region Countries

Table E: Country schedules and modelled routine immunisation coverages: South East Asia region countries.

Country	Age at first dose	Age at second dose	Modelled Coverage			Second dose introduced
	(weeks)	(weeks)	6 weeks	14 weeks	39 weeks	
Bangladesh	6	14	99%	98%	97%	2017
Bhutan	14	32	96%	96%	96%	2021
Indonesia	17	39	85%	82%	70%	2022
Maldives	26		99%	99%	99%	
Myanmar	17		69%	69%	69%	
Nepal	14	39	94%	93%	90%	2017
Sri Lanka	9	17	99%	99%	99%	2016
Thailand	9	17	94%	93%	90%	2023
Timor-Leste	14		83%	82%	77%	

1.6 Western Pacific Region Countries

Table F: Country schedules and modelled routine immunisation coverages: Western Pacific region countries.

Country	Age at first dose	Age at second dose	Modelled Coverage			Second dose introduced
	(weeks)	(weeks)	6 weeks	14 weeks	39 weeks	
Cambodia	14		87%	85%	78%	
China	9	13	98%	98%	97%	2019
Cook Islands	22		96%	95%	87%	

Fiji	14	52	99%	99%	98%	2024
Kiribati	14	52	94%	93%	92%	2024
Lao People's Democratic Republic	14	52	85%	83%	76%	2024
Mongolia	22		98%	98%	97%	
Nauru	14		98%	98%	98%	
Papua New Guinea	13	39	39%	39%	39%	2021
Philippines	14	39	85%	83%	78%	2021
Samoa	14		95%	94%	87%	
Solomon Islands	14		84%	81%	71%	
Tonga	14		99%	99%	99%	
Vanuatu	14		78%	76%	69%	
Viet Nam	22	39	84%	84%	83%	2022

2 Immunity projections

Here we display the projections of immunity in children under the age of five 2024-2031, assuming 2023 modelled coverages are maintained, together with the effects of switching to the standard fourteen + thirty-nine week schedule for children born in January 2025 onwards, and subsequent catch-ups, with coverage of either 80% or 50% of children who had received fewer than two doses.

2.1 African Region countries

Table G: IPV-induced immunity projections under different schedules and scenarios, for African region countries using one or two doses of IPV.

Country	Scenario	2024	2025	2026	2027	2028	2029	2030	2031
Angola	Current Schedule: 2 doses	37%	39%	41%	44%	46%	46%	46%	46%
	14/39 by 2026	37%	39%	41%	44%	45%	45%	45%	45%
	14/39 by 2029	37%	39%	41%	44%	46%	46%	46%	45%
	14/39 by 2026, 50% catchup Jan 2027	37%	39%	41%	65%	61%	56%	51%	46%
	14/39 by 2026, 80% catchup Jan 2027	37%	39%	41%	78%	71%	63%	55%	47%
	14/39 by 2026, 50% catchup Jan 2030	37%	39%	41%	44%	45%	45%	66%	61%
	14/39 by 2026, 80% catchup Jan 2030	37%	39%	41%	44%	45%	45%	78%	70%
Benin	Current Schedule: 1 dose	48%	48%	48%	48%	49%	49%	49%	49%
	14/39 by 2026	48%	48%	48%	49%	52%	56%	59%	62%
	14/39 by 2029	48%	48%	48%	48%	49%	49%	49%	52%
	14/39 by 2026, 50% catchup Jan 2027	48%	48%	48%	69%	67%	66%	64%	63%
	14/39 by 2026, 80% catchup Jan 2027	48%	48%	48%	81%	76%	72%	68%	63%
	14/39 by 2026, 50% catchup Jan 2030	48%	48%	48%	49%	52%	56%	74%	72%
	14/39 by 2026, 80% catchup Jan 2030	48%	48%	48%	49%	52%	56%	83%	78%
Botswana	Current Schedule: 2 doses	68%	67%	69%	73%	77%	80%	82%	82%
	14/39 by 2026	68%	67%	69%	73%	77%	80%	81%	81%
	14/39 by 2029	68%	67%	69%	73%	77%	80%	82%	82%
	14/39 by 2026, 50% catchup Jan 2027	68%	67%	69%	82%	82%	83%	83%	81%
	14/39 by 2026, 80% catchup Jan 2027	68%	67%	69%	87%	86%	85%	83%	82%
	14/39 by 2026, 50% catchup Jan 2030	68%	67%	69%	73%	77%	80%	86%	85%
	14/39 by 2026, 80% catchup Jan 2030	68%	67%	69%	73%	77%	80%	89%	87%
Burkina Faso	Current Schedule: 2 doses	73%	77%	81%	85%	85%	85%	85%	85%
	14/39 by 2026	73%	77%	81%	85%	85%	85%	85%	85%
	14/39 by 2029	73%	77%	81%	85%	85%	85%	85%	85%

	14/39 by 2026, 50% catchup Jan 2027	73%	77%	81%	88%	87%	86%	86%	85%
	14/39 by 2026, 80% catchup Jan 2027	73%	77%	81%	90%	89%	87%	86%	85%
	14/39 by 2026, 50% catchup Jan 2030	73%	77%	81%	85%	85%	85%	88%	87%
	14/39 by 2026, 80% catchup Jan 2030	73%	77%	81%	85%	85%	85%	90%	88%
Burundi	Current Schedule: 1 dose	60%	59%	58%	58%	57%	57%	57%	57%
	14/39 by 2026	60%	59%	58%	59%	64%	70%	75%	81%
	14/39 by 2029	60%	59%	58%	58%	57%	57%	59%	64%
	14/39 by 2026, 50% catchup Jan 2027	60%	59%	58%	75%	76%	78%	79%	81%
	14/39 by 2026, 80% catchup Jan 2027	60%	59%	58%	84%	83%	82%	82%	81%
	14/39 by 2026, 50% catchup Jan 2030	60%	59%	58%	59%	64%	70%	83%	84%
	14/39 by 2026, 80% catchup Jan 2030	60%	59%	58%	59%	64%	70%	87%	86%
Cabo Verde	Current Schedule: 2 doses	70%	74%	78%	82%	86%	86%	86%	86%
	14/39 by 2026	70%	74%	78%	82%	86%	86%	86%	86%
	14/39 by 2029	70%	74%	78%	82%	86%	86%	86%	86%
	14/39 by 2026, 50% catchup Jan 2027	70%	74%	78%	86%	88%	88%	87%	86%
	14/39 by 2026, 80% catchup Jan 2027	70%	74%	78%	89%	89%	88%	87%	86%
	14/39 by 2026, 50% catchup Jan 2030	70%	74%	78%	82%	86%	86%	89%	88%
	14/39 by 2026, 80% catchup Jan 2030	70%	74%	78%	82%	86%	86%	90%	89%
Cameroon	Current Schedule: 2 doses	48%	52%	57%	62%	66%	66%	66%	66%
	14/39 by 2026	48%	52%	57%	62%	66%	66%	66%	66%
	14/39 by 2029	48%	52%	57%	62%	66%	66%	66%	66%
	14/39 by 2026, 50% catchup Jan 2027	48%	52%	57%	75%	75%	72%	69%	67%
	14/39 by 2026, 80% catchup Jan 2027	48%	52%	57%	84%	80%	76%	72%	67%
	14/39 by 2026, 50% catchup Jan 2030	48%	52%	57%	62%	66%	66%	78%	75%
	14/39 by 2026, 80% catchup Jan 2030	48%	52%	57%	62%	66%	66%	84%	80%
Central African Republic	Current Schedule: 1 dose	31%	31%	31%	31%	31%	31%	31%	31%
	14/39 by 2026	31%	31%	31%	32%	34%	36%	39%	41%
	14/39 by 2029	31%	31%	31%	31%	31%	31%	32%	34%
	14/39 by 2026, 50% catchup Jan 2027	31%	31%	31%	59%	55%	51%	46%	42%
	14/39 by 2026, 80% catchup Jan 2027	31%	31%	31%	75%	67%	59%	51%	43%
	14/39 by 2026, 50% catchup Jan 2030	31%	31%	31%	32%	34%	36%	62%	58%
	14/39 by 2026, 80% catchup Jan 2030	31%	31%	31%	32%	34%	36%	76%	68%
Chad	Current Schedule: 2 doses	52%	57%	61%	62%	62%	62%	62%	62%
	14/39 by 2026	52%	57%	61%	62%	62%	62%	62%	62%
	14/39 by 2029	52%	57%	61%	62%	62%	62%	62%	62%
	14/39 by 2026, 50% catchup Jan 2027	52%	57%	61%	75%	72%	69%	66%	63%
	14/39 by 2026, 80% catchup Jan 2027	52%	57%	61%	83%	78%	73%	68%	63%
	14/39 by 2026, 50% catchup Jan 2030	52%	57%	61%	62%	62%	62%	75%	72%
	14/39 by 2026, 80% catchup Jan 2030	52%	57%	61%	62%	62%	62%	83%	78%
Comoros	Current Schedule: 2 doses	51%	51%	52%	57%	62%	67%	72%	72%
	14/39 by 2026	51%	51%	52%	57%	62%	67%	72%	72%
	14/39 by 2029	51%	51%	52%	57%	62%	67%	72%	72%
	14/39 by 2026, 50% catchup Jan 2027	51%	51%	52%	73%	73%	74%	74%	72%
	14/39 by 2026, 80% catchup Jan 2027	51%	51%	52%	83%	80%	78%	76%	72%
	14/39 by 2026, 50% catchup Jan 2030	51%	51%	52%	57%	62%	67%	80%	78%
	14/39 by 2026, 80% catchup Jan 2030	51%	51%	52%	57%	62%	67%	86%	82%
Congo	Current Schedule: 2 doses	50%	50%	50%	52%	55%	58%	61%	61%
	14/39 by 2026	50%	50%	50%	52%	55%	58%	60%	61%

	14/39 by 2029	50%	50%	50%	52%	55%	58%	61%	61%
	14/39 by 2026, 50% catchup Jan 2027	50%	50%	50%	70%	68%	66%	64%	61%
	14/39 by 2026, 80% catchup Jan 2027	50%	50%	50%	81%	76%	72%	67%	62%
	14/39 by 2026, 50% catchup Jan 2030	50%	50%	50%	52%	55%	58%	74%	71%
	14/39 by 2026, 80% catchup Jan 2030	50%	50%	50%	52%	55%	58%	83%	78%
Cote d'Ivoire	Current Schedule: 2 doses	49%	50%	51%	53%	54%	54%	54%	54%
	14/39 by 2026	49%	50%	51%	52%	56%	59%	61%	63%
	14/39 by 2029	49%	50%	51%	53%	54%	54%	54%	56%
	14/39 by 2026, 50% catchup Jan 2027	49%	50%	51%	66%	65%	65%	64%	64%
	14/39 by 2026, 80% catchup Jan 2027	49%	50%	51%	75%	71%	68%	66%	65%
	14/39 by 2026, 50% catchup Jan 2030	49%	50%	51%	52%	56%	59%	73%	73%
	14/39 by 2026, 80% catchup Jan 2030	49%	50%	51%	52%	56%	59%	81%	79%
Democratic Republic of the Congo	Current Schedule: 2 doses	45%	47%	50%	53%	56%	56%	56%	56%
	14/39 by 2026	45%	47%	50%	53%	56%	56%	56%	56%
	14/39 by 2029	45%	47%	50%	53%	56%	56%	56%	56%
	14/39 by 2026, 50% catchup Jan 2027	45%	47%	50%	70%	68%	64%	60%	56%
	14/39 by 2026, 80% catchup Jan 2027	45%	47%	50%	81%	75%	69%	63%	57%
	14/39 by 2026, 50% catchup Jan 2030	45%	47%	50%	53%	56%	56%	72%	68%
	14/39 by 2026, 80% catchup Jan 2030	45%	47%	50%	53%	56%	56%	81%	75%
Equatorial Guinea	Current Schedule: 1 dose	47%	48%	49%	50%	50%	50%	50%	50%
	14/39 by 2026	47%	48%	49%	51%	54%	57%	60%	62%
	14/39 by 2029	47%	48%	49%	50%	50%	50%	51%	54%
	14/39 by 2026, 50% catchup Jan 2027	47%	48%	49%	70%	68%	67%	65%	63%
	14/39 by 2026, 80% catchup Jan 2027	47%	48%	49%	81%	77%	72%	68%	64%
	14/39 by 2026, 50% catchup Jan 2030	47%	48%	49%	51%	54%	57%	74%	73%
	14/39 by 2026, 80% catchup Jan 2030	47%	48%	49%	51%	54%	57%	83%	79%
Eritrea	Current Schedule: 2 doses	72%	78%	84%	87%	87%	87%	87%	87%
	14/39 by 2026	72%	78%	84%	87%	87%	87%	87%	87%
	14/39 by 2029	72%	78%	84%	87%	87%	87%	87%	87%
	14/39 by 2026, 50% catchup Jan 2027	72%	78%	84%	89%	88%	88%	87%	87%
	14/39 by 2026, 80% catchup Jan 2027	72%	78%	84%	90%	89%	88%	88%	87%
	14/39 by 2026, 50% catchup Jan 2030	72%	78%	84%	87%	87%	87%	89%	88%
	14/39 by 2026, 80% catchup Jan 2030	72%	78%	84%	87%	87%	87%	90%	89%
Eswatini	Current Schedule: 2 doses	56%	54%	56%	60%	64%	70%	74%	74%
	14/39 by 2026	56%	54%	56%	60%	64%	70%	74%	74%
	14/39 by 2029	56%	54%	56%	60%	64%	70%	74%	74%
	14/39 by 2026, 50% catchup Jan 2027	56%	54%	56%	75%	75%	76%	76%	75%
	14/39 by 2026, 80% catchup Jan 2027	56%	54%	56%	83%	81%	80%	78%	75%
	14/39 by 2026, 50% catchup Jan 2030	56%	54%	56%	60%	64%	70%	82%	80%
	14/39 by 2026, 80% catchup Jan 2030	56%	54%	56%	60%	64%	70%	86%	83%
Ethiopia	Current Schedule: 2 doses	44%	45%	49%	53%	57%	61%	61%	61%
	14/39 by 2026	44%	45%	49%	53%	57%	61%	61%	61%
	14/39 by 2029	44%	45%	49%	53%	57%	61%	61%	61%
	14/39 by 2026, 50% catchup Jan 2027	44%	45%	49%	71%	69%	68%	65%	62%
	14/39 by 2026, 80% catchup Jan 2027	44%	45%	49%	81%	77%	72%	67%	62%
	14/39 by 2026, 50% catchup Jan 2030	44%	45%	49%	53%	57%	61%	75%	71%
	14/39 by 2026, 80% catchup Jan 2030	44%	45%	49%	53%	57%	61%	83%	78%
Gabon	Current Schedule: 1 dose	46%	46%	46%	46%	47%	47%	47%	47%

	14/39 by 2026	46%	46%	46%	47%	51%	54%	57%	60%
	14/39 by 2029	46%	46%	46%	46%	47%	47%	48%	51%
	14/39 by 2026, 50% catchup Jan 2027	46%	46%	46%	68%	66%	65%	63%	61%
	14/39 by 2026, 80% catchup Jan 2027	46%	46%	46%	80%	76%	71%	66%	61%
	14/39 by 2026, 50% catchup Jan 2030	46%	46%	46%	47%	51%	54%	73%	71%
	14/39 by 2026, 80% catchup Jan 2030	46%	46%	46%	47%	51%	54%	82%	77%
Gambia	Current Schedule: 2 doses	61%	62%	65%	69%	69%	69%	69%	69%
	14/39 by 2026	61%	62%	65%	68%	69%	69%	68%	68%
	14/39 by 2029	61%	62%	65%	69%	69%	69%	69%	69%
	14/39 by 2026, 50% catchup Jan 2027	61%	62%	65%	79%	76%	74%	71%	69%
	14/39 by 2026, 80% catchup Jan 2027	61%	62%	65%	85%	81%	77%	73%	69%
	14/39 by 2026, 50% catchup Jan 2030	61%	62%	65%	68%	69%	69%	79%	76%
	14/39 by 2026, 80% catchup Jan 2030	61%	62%	65%	68%	69%	69%	85%	81%
Ghana	Current Schedule: 2 doses	62%	62%	65%	70%	76%	82%	87%	87%
	14/39 by 2026	62%	62%	65%	70%	76%	82%	87%	87%
	14/39 by 2029	62%	62%	65%	70%	76%	82%	87%	87%
	14/39 by 2026, 50% catchup Jan 2027	62%	62%	65%	81%	83%	85%	87%	87%
	14/39 by 2026, 80% catchup Jan 2027	62%	62%	65%	87%	87%	87%	88%	87%
	14/39 by 2026, 50% catchup Jan 2030	62%	62%	65%	70%	76%	82%	89%	88%
	14/39 by 2026, 80% catchup Jan 2030	62%	62%	65%	70%	76%	82%	90%	89%
Guinea	Current Schedule: 1 dose	36%	36%	36%	36%	36%	36%	36%	36%
	14/39 by 2026	36%	36%	36%	37%	39%	42%	44%	47%
	14/39 by 2029	36%	36%	36%	36%	36%	36%	37%	39%
	14/39 by 2026, 50% catchup Jan 2027	36%	36%	36%	62%	58%	55%	52%	48%
	14/39 by 2026, 80% catchup Jan 2027	36%	36%	36%	77%	70%	63%	56%	49%
	14/39 by 2026, 50% catchup Jan 2030	36%	36%	36%	37%	39%	42%	66%	62%
	14/39 by 2026, 80% catchup Jan 2030	36%	36%	36%	37%	39%	42%	78%	71%
Guinea-Bissau	Current Schedule: 1 dose	48%	47%	48%	50%	50%	50%	50%	50%
	14/39 by 2026	48%	47%	48%	51%	55%	60%	64%	69%
	14/39 by 2029	48%	47%	48%	50%	50%	50%	51%	55%
	14/39 by 2026, 50% catchup Jan 2027	48%	47%	48%	70%	70%	69%	69%	69%
	14/39 by 2026, 80% catchup Jan 2027	48%	47%	48%	81%	78%	75%	72%	69%
	14/39 by 2026, 50% catchup Jan 2030	48%	47%	48%	51%	55%	60%	76%	76%
	14/39 by 2026, 80% catchup Jan 2030	48%	47%	48%	51%	55%	60%	84%	81%
Kenya	Current Schedule: 1 dose	60%	60%	59%	59%	59%	59%	59%	59%
	14/39 by 2026	60%	60%	59%	60%	65%	70%	76%	81%
	14/39 by 2029	60%	60%	59%	59%	59%	59%	60%	65%
	14/39 by 2026, 50% catchup Jan 2027	60%	60%	59%	75%	77%	78%	80%	81%
	14/39 by 2026, 80% catchup Jan 2027	60%	60%	59%	84%	83%	83%	82%	81%
	14/39 by 2026, 50% catchup Jan 2030	60%	60%	59%	60%	65%	70%	83%	84%
	14/39 by 2026, 80% catchup Jan 2030	60%	60%	59%	60%	65%	70%	87%	87%
Lesotho	Current Schedule: 2 doses	57%	56%	58%	62%	67%	72%	77%	77%
	14/39 by 2026	57%	56%	58%	62%	67%	72%	77%	77%
	14/39 by 2029	57%	56%	58%	62%	67%	72%	77%	77%
	14/39 by 2026, 50% catchup Jan 2027	57%	56%	58%	76%	77%	78%	79%	77%
	14/39 by 2026, 80% catchup Jan 2027	57%	56%	58%	84%	83%	81%	80%	77%
	14/39 by 2026, 50% catchup Jan 2030	57%	56%	58%	62%	67%	72%	83%	82%
	14/39 by 2026, 80% catchup Jan 2030	57%	56%	58%	62%	67%	72%	87%	85%

	Current Schedule: 1 dose	50%	52%	54%	56%	57%	57%	57%	57%
	14/39 by 2026	50%	52%	54%	57%	63%	67%	72%	76%
	14/39 by 2029	50%	52%	54%	56%	57%	57%	58%	63%
	14/39 by 2026, 50% catchup Jan 2027	50%	52%	54%	74%	75%	75%	76%	77%
	14/39 by 2026, 80% catchup Jan 2027	50%	52%	54%	83%	82%	80%	79%	77%
	14/39 by 2026, 50% catchup Jan 2030	50%	52%	54%	57%	63%	67%	81%	81%
	14/39 by 2026, 80% catchup Jan 2030	50%	52%	54%	57%	63%	67%	86%	85%
Madagascar	Current Schedule: 2 doses	46%	48%	51%	53%	54%	54%	54%	54%
	14/39 by 2026	46%	48%	51%	53%	54%	54%	54%	54%
	14/39 by 2029	46%	48%	51%	53%	54%	54%	54%	54%
	14/39 by 2026, 50% catchup Jan 2027	46%	48%	51%	70%	67%	63%	59%	55%
	14/39 by 2026, 80% catchup Jan 2027	46%	48%	51%	81%	75%	68%	62%	55%
	14/39 by 2026, 50% catchup Jan 2030	46%	48%	51%	53%	54%	54%	71%	67%
	14/39 by 2026, 80% catchup Jan 2030	46%	48%	51%	53%	54%	54%	81%	75%
Malawi	Current Schedule: 2 doses	59%	58%	60%	65%	70%	75%	79%	79%
	14/39 by 2026	59%	58%	60%	65%	70%	75%	79%	79%
	14/39 by 2029	59%	58%	60%	65%	70%	75%	79%	79%
	14/39 by 2026, 50% catchup Jan 2027	59%	58%	60%	77%	79%	80%	81%	79%
	14/39 by 2026, 80% catchup Jan 2027	59%	58%	60%	85%	84%	83%	82%	80%
	14/39 by 2026, 50% catchup Jan 2030	59%	58%	60%	65%	70%	75%	85%	83%
	14/39 by 2026, 80% catchup Jan 2030	59%	58%	60%	65%	70%	75%	88%	86%
Mali	Current Schedule: 2 doses	56%	60%	64%	63%	63%	63%	63%	63%
	14/39 by 2026	56%	60%	64%	63%	63%	63%	63%	63%
	14/39 by 2029	56%	60%	64%	63%	63%	63%	63%	63%
	14/39 by 2026, 50% catchup Jan 2027	56%	60%	64%	76%	73%	70%	67%	64%
	14/39 by 2026, 80% catchup Jan 2027	56%	60%	64%	83%	79%	74%	69%	64%
	14/39 by 2026, 50% catchup Jan 2030	56%	60%	64%	63%	63%	63%	76%	73%
	14/39 by 2026, 80% catchup Jan 2030	56%	60%	64%	63%	63%	63%	83%	79%
Mauritania	Current Schedule: 1 dose	56%	57%	59%	60%	61%	61%	61%	61%
	14/39 by 2026	56%	57%	59%	61%	66%	71%	75%	80%
	14/39 by 2029	56%	57%	59%	60%	61%	61%	62%	66%
	14/39 by 2026, 50% catchup Jan 2027	56%	57%	59%	76%	77%	78%	79%	80%
	14/39 by 2026, 80% catchup Jan 2027	56%	57%	59%	85%	84%	83%	81%	80%
	14/39 by 2026, 50% catchup Jan 2030	56%	57%	59%	61%	66%	71%	83%	84%
	14/39 by 2026, 80% catchup Jan 2030	56%	57%	59%	61%	66%	71%	88%	86%
Mozambique	Current Schedule: 2 doses	55%	56%	60%	64%	67%	69%	69%	69%
	14/39 by 2026	55%	56%	60%	64%	67%	69%	68%	68%
	14/39 by 2029	55%	56%	60%	64%	67%	69%	69%	69%
	14/39 by 2026, 50% catchup Jan 2027	55%	56%	60%	77%	76%	74%	71%	69%
	14/39 by 2026, 80% catchup Jan 2027	55%	56%	60%	84%	81%	77%	73%	69%
	14/39 by 2026, 50% catchup Jan 2030	55%	56%	60%	64%	67%	69%	79%	76%
	14/39 by 2026, 80% catchup Jan 2030	55%	56%	60%	64%	67%	69%	85%	81%
Namibia	Current Schedule: 2 doses	57%	62%	66%	70%	75%	75%	75%	75%
	14/39 by 2026	57%	62%	66%	70%	75%	75%	75%	75%
	14/39 by 2029	57%	62%	66%	70%	75%	75%	75%	75%
	14/39 by 2026, 50% catchup Jan 2027	57%	62%	66%	80%	81%	79%	78%	76%
	14/39 by 2026, 80% catchup Jan 2027	57%	62%	66%	86%	84%	81%	79%	76%
	14/39 by 2026, 50% catchup Jan 2030	57%	62%	66%	70%	75%	75%	83%	81%

	14/39 by 2026, 80% catchup Jan 2030	57%	62%	66%	70%	75%	75%	87%	84%
Niger	Current Schedule: 2 doses	58%	63%	68%	72%	76%	76%	76%	76%
	14/39 by 2026	58%	63%	68%	72%	76%	76%	76%	76%
	14/39 by 2029	58%	63%	68%	72%	76%	76%	76%	76%
	14/39 by 2026, 50% catchup Jan 2027	58%	63%	68%	81%	82%	80%	78%	77%
	14/39 by 2026, 80% catchup Jan 2027	58%	63%	68%	87%	85%	82%	79%	77%
	14/39 by 2026, 50% catchup Jan 2030	58%	63%	68%	72%	76%	76%	83%	82%
	14/39 by 2026, 80% catchup Jan 2030	58%	63%	68%	72%	76%	76%	87%	85%
Nigeria	Current Schedule: 2 doses	43%	43%	44%	44%	44%	44%	44%	44%
	14/39 by 2026	43%	43%	44%	44%	46%	48%	50%	52%
	14/39 by 2029	43%	43%	44%	44%	44%	44%	44%	46%
	14/39 by 2026, 50% catchup Jan 2027	43%	43%	44%	60%	58%	56%	55%	53%
	14/39 by 2026, 80% catchup Jan 2027	43%	43%	44%	70%	66%	62%	58%	54%
	14/39 by 2026, 50% catchup Jan 2030	43%	43%	44%	44%	46%	48%	67%	65%
	14/39 by 2026, 80% catchup Jan 2030	43%	43%	44%	44%	46%	48%	77%	73%
Rwanda	Current Schedule: 2 doses	67%	73%	80%	86%	87%	87%	87%	87%
	14/39 by 2026	67%	73%	80%	86%	87%	87%	87%	87%
	14/39 by 2029	67%	73%	80%	86%	87%	87%	87%	87%
	14/39 by 2026, 50% catchup Jan 2027	67%	73%	80%	89%	89%	88%	88%	87%
	14/39 by 2026, 80% catchup Jan 2027	67%	73%	80%	90%	90%	89%	88%	88%
	14/39 by 2026, 50% catchup Jan 2030	67%	73%	80%	86%	87%	87%	89%	89%
	14/39 by 2026, 80% catchup Jan 2030	67%	73%	80%	86%	87%	87%	90%	90%
Sao Tome and Principe	Current Schedule: 2 doses	58%	56%	59%	63%	68%	73%	75%	75%
	14/39 by 2026	58%	56%	59%	63%	68%	73%	75%	75%
	14/39 by 2029	58%	56%	59%	63%	68%	73%	75%	75%
	14/39 by 2026, 50% catchup Jan 2027	58%	56%	59%	76%	77%	78%	77%	75%
	14/39 by 2026, 80% catchup Jan 2027	58%	56%	59%	84%	82%	81%	78%	75%
	14/39 by 2026, 50% catchup Jan 2030	58%	56%	59%	63%	68%	73%	82%	80%
	14/39 by 2026, 80% catchup Jan 2030	58%	56%	59%	63%	68%	73%	86%	84%
Senegal	Current Schedule: 2 doses	60%	60%	61%	62%	64%	64%	64%	64%
	14/39 by 2026	60%	60%	61%	61%	66%	69%	72%	75%
	14/39 by 2029	60%	60%	61%	62%	64%	64%	63%	66%
	14/39 by 2026, 50% catchup Jan 2027	60%	60%	61%	69%	70%	72%	74%	76%
	14/39 by 2026, 80% catchup Jan 2027	60%	60%	61%	74%	73%	74%	75%	76%
	14/39 by 2026, 50% catchup Jan 2030	60%	60%	61%	61%	66%	69%	79%	81%
	14/39 by 2026, 80% catchup Jan 2030	60%	60%	61%	61%	66%	69%	83%	84%
Sierra Leone	Current Schedule: 2 doses	66%	71%	76%	78%	78%	78%	78%	78%
	14/39 by 2026	66%	71%	76%	78%	78%	78%	78%	78%
	14/39 by 2029	66%	71%	76%	78%	78%	78%	78%	78%
	14/39 by 2026, 50% catchup Jan 2027	66%	71%	76%	84%	83%	81%	80%	79%
	14/39 by 2026, 80% catchup Jan 2027	66%	71%	76%	88%	86%	83%	81%	79%
	14/39 by 2026, 50% catchup Jan 2030	66%	71%	76%	78%	78%	78%	84%	83%
	14/39 by 2026, 80% catchup Jan 2030	66%	71%	76%	78%	78%	78%	88%	86%
South Sudan	Current Schedule: 2 doses	50%	54%	58%	59%	59%	59%	59%	59%
	14/39 by 2026	50%	54%	58%	59%	59%	59%	59%	59%
	14/39 by 2029	50%	54%	58%	59%	59%	59%	59%	59%
	14/39 by 2026, 50% catchup Jan 2027	50%	54%	58%	74%	70%	67%	63%	60%
	14/39 by 2026, 80% catchup Jan 2027	50%	54%	58%	82%	77%	71%	66%	61%

	14/39 by 2026, 50% catchup Jan 2030	50%	54%	58%	59%	59%	59%	74%	70%
	14/39 by 2026, 80% catchup Jan 2030	50%	54%	58%	59%	59%	59%	82%	77%
Togo	Current Schedule: 2 doses	58%	62%	67%	71%	74%	74%	74%	74%
	14/39 by 2026	58%	62%	67%	71%	74%	74%	74%	74%
	14/39 by 2029	58%	62%	67%	71%	74%	74%	74%	74%
	14/39 by 2026, 50% catchup Jan 2027	58%	62%	67%	80%	80%	78%	77%	75%
	14/39 by 2026, 80% catchup Jan 2027	58%	62%	67%	86%	84%	81%	78%	75%
	14/39 by 2026, 50% catchup Jan 2030	58%	62%	67%	71%	74%	74%	82%	80%
	14/39 by 2026, 80% catchup Jan 2030	58%	62%	67%	71%	74%	74%	87%	84%
Uganda	Current Schedule: 2 doses	62%	63%	65%	66%	67%	67%	67%	67%
	14/39 by 2026	62%	63%	65%	65%	70%	74%	78%	81%
	14/39 by 2029	62%	63%	65%	66%	67%	67%	67%	70%
	14/39 by 2026, 50% catchup Jan 2027	62%	63%	65%	71%	73%	76%	79%	82%
	14/39 by 2026, 80% catchup Jan 2027	62%	63%	65%	74%	74%	77%	79%	82%
	14/39 by 2026, 50% catchup Jan 2030	62%	63%	65%	65%	70%	74%	82%	85%
	14/39 by 2026, 80% catchup Jan 2030	62%	63%	65%	65%	70%	74%	84%	87%
United Republic of Tanzania	Current Schedule: 2 doses	59%	59%	60%	66%	71%	77%	82%	84%
	14/39 by 2026	59%	59%	60%	66%	71%	77%	82%	84%
	14/39 by 2029	59%	59%	60%	66%	71%	77%	82%	84%
	14/39 by 2026, 50% catchup Jan 2027	59%	59%	60%	78%	80%	82%	84%	84%
	14/39 by 2026, 80% catchup Jan 2027	59%	59%	60%	86%	85%	85%	85%	84%
	14/39 by 2026, 50% catchup Jan 2030	59%	59%	60%	66%	71%	77%	86%	86%
	14/39 by 2026, 80% catchup Jan 2030	59%	59%	60%	66%	71%	77%	89%	88%
Zambia	Current Schedule: 2 doses	54%	52%	52%	55%	60%	65%	69%	69%
	14/39 by 2026	54%	52%	52%	55%	60%	65%	69%	69%
	14/39 by 2029	54%	52%	52%	55%	60%	65%	69%	69%
	14/39 by 2026, 50% catchup Jan 2027	54%	52%	52%	72%	72%	72%	72%	69%
	14/39 by 2026, 80% catchup Jan 2027	54%	52%	52%	82%	79%	76%	73%	70%
	14/39 by 2026, 50% catchup Jan 2030	54%	52%	52%	55%	60%	65%	79%	76%
	14/39 by 2026, 80% catchup Jan 2030	54%	52%	52%	55%	60%	65%	85%	81%
Zimbabwe	Current Schedule: 2 doses	66%	72%	77%	81%	81%	81%	81%	81%
	14/39 by 2026	66%	72%	77%	81%	81%	81%	81%	81%
	14/39 by 2029	66%	72%	77%	81%	81%	81%	81%	81%
	14/39 by 2026, 50% catchup Jan 2027	66%	72%	77%	85%	84%	83%	82%	81%
	14/39 by 2026, 80% catchup Jan 2027	66%	72%	77%	88%	86%	85%	83%	81%
	14/39 by 2026, 50% catchup Jan 2030	66%	72%	77%	81%	81%	81%	85%	84%
	14/39 by 2026, 80% catchup Jan 2030	66%	72%	77%	81%	81%	81%	88%	86%

2.2 Americas Region countries

Table H: IPV-induced immunity projections under different schedules and scenarios, for Americas region countries using one or two doses of IPV.

Country	Scenario	2024	2025	2026	2027	2028	2029	2030	2031
Anguilla	Current Schedule: 2 doses	78%	79%	79%	79%	79%	79%	79%	79%
	14/39 by 2026	78%	79%	79%	78%	80%	82%	84%	86%
	14/39 by 2029	78%	79%	79%	79%	79%	79%	78%	80%
	14/39 by 2026, 50% catchup Jan 2027	78%	79%	79%	79%	81%	83%	84%	86%

	14/39 by 2026, 80% catchup Jan 2027	78%	79%	79%	80%	82%	83%	85%	86%
	14/39 by 2026, 50% catchup Jan 2030	78%	79%	79%	78%	80%	82%	86%	88%
	14/39 by 2026, 80% catchup Jan 2030	78%	79%	79%	78%	80%	82%	88%	89%
Antigua and Barbuda	Current Schedule: 2 doses	77%	76%	75%	75%	75%	75%	75%	75%
	14/39 by 2026	77%	76%	75%	74%	77%	80%	82%	85%
	14/39 by 2029	77%	76%	75%	75%	75%	75%	74%	77%
	14/39 by 2026, 50% catchup Jan 2027	77%	76%	75%	78%	80%	82%	83%	85%
	14/39 by 2026, 80% catchup Jan 2027	77%	76%	75%	80%	82%	83%	84%	85%
	14/39 by 2026, 50% catchup Jan 2030	77%	76%	75%	74%	77%	80%	85%	87%
	14/39 by 2026, 80% catchup Jan 2030	77%	76%	75%	74%	77%	80%	87%	89%
Bahamas	Current Schedule: 2 doses	71%	70%	69%	69%	69%	69%	69%	69%
	14/39 by 2026	71%	70%	69%	68%	70%	72%	74%	75%
14/39 by 2029	71%	70%	69%	69%	69%	69%	68%	70%	
	14/39 by 2026, 50% catchup Jan 2027	71%	70%	69%	75%	75%	75%	76%	76%
	14/39 by 2026, 80% catchup Jan 2027	71%	70%	69%	79%	78%	77%	77%	76%
	14/39 by 2026, 50% catchup Jan 2030	71%	70%	69%	68%	70%	72%	81%	81%
	14/39 by 2026, 80% catchup Jan 2030	71%	70%	69%	68%	70%	72%	85%	84%
Barbados	Current Schedule: 2 doses	72%	72%	72%	73%	73%	73%	73%	73%
	14/39 by 2026	72%	72%	72%	72%	74%	76%	78%	80%
	14/39 by 2029	72%	72%	72%	73%	73%	73%	72%	74%
	14/39 by 2026, 50% catchup Jan 2027	72%	72%	72%	77%	78%	79%	80%	81%
	14/39 by 2026, 80% catchup Jan 2027	72%	72%	72%	79%	80%	80%	81%	81%
	14/39 by 2026, 50% catchup Jan 2030	72%	72%	72%	72%	74%	76%	83%	84%
	14/39 by 2026, 80% catchup Jan 2030	72%	72%	72%	72%	74%	76%	86%	87%
Belize	Current Schedule: 2 doses	63%	70%	71%	72%	72%	72%	72%	72%
	14/39 by 2026	63%	70%	71%	71%	72%	74%	76%	78%
	14/39 by 2029	63%	70%	71%	72%	72%	72%	70%	72%
	14/39 by 2026, 50% catchup Jan 2027	63%	70%	71%	76%	76%	77%	78%	79%
	14/39 by 2026, 80% catchup Jan 2027	63%	70%	71%	79%	79%	79%	79%	79%
	14/39 by 2026, 50% catchup Jan 2030	63%	70%	71%	71%	72%	74%	82%	83%
	14/39 by 2026, 80% catchup Jan 2030	63%	70%	71%	71%	72%	74%	85%	85%
Bolivia (Plurinational State of)	Current Schedule: 2 doses	49%	56%	63%	64%	65%	65%	65%	65%
	14/39 by 2026	49%	56%	63%	63%	64%	64%	64%	64%
	14/39 by 2029	49%	56%	63%	64%	65%	65%	64%	64%
	14/39 by 2026, 50% catchup Jan 2027	49%	56%	63%	74%	73%	70%	67%	65%
	14/39 by 2026, 80% catchup Jan 2027	49%	56%	63%	81%	78%	74%	69%	65%
	14/39 by 2026, 50% catchup Jan 2030	49%	56%	63%	63%	64%	64%	76%	73%
	14/39 by 2026, 80% catchup Jan 2030	49%	56%	63%	63%	64%	64%	83%	79%
British Virgin Islands	Current Schedule: 2 doses	78%	80%	82%	83%	82%	82%	82%	82%
	14/39 by 2026	78%	80%	82%	82%	80%	80%	79%	79%
	14/39 by 2029	78%	80%	82%	83%	82%	82%	81%	80%
	14/39 by 2026, 50% catchup Jan 2027	78%	80%	82%	85%	83%	82%	80%	79%
	14/39 by 2026, 80% catchup Jan 2027	78%	80%	82%	87%	85%	83%	81%	79%
	14/39 by 2026, 50% catchup Jan 2030	78%	80%	82%	82%	80%	80%	84%	83%
	14/39 by 2026, 80% catchup Jan 2030	78%	80%	82%	82%	80%	80%	87%	86%
Cuba	Current Schedule: 2 doses	88%	88%	88%	88%	88%	88%	88%	88%
	14/39 by 2026	88%	88%	88%	87%	87%	87%	87%	87%
	14/39 by 2029	88%	88%	88%	88%	88%	88%	87%	87%

	14/39 by 2026, 50% catchup Jan 2027	88%	88%	88%	87%	87%	87%	87%	87%
	14/39 by 2026, 80% catchup Jan 2027	88%	88%	88%	87%	87%	87%	87%	87%
	14/39 by 2026, 50% catchup Jan 2030	88%	88%	88%	87%	87%	87%	88%	88%
	14/39 by 2026, 80% catchup Jan 2030	88%	88%	88%	87%	87%	87%	88%	88%
Dominica	Current Schedule: 2 doses	61%	67%	74%	73%	72%	72%	72%	72%
	14/39 by 2026	61%	67%	74%	71%	73%	75%	77%	79%
	14/39 by 2029	61%	67%	74%	73%	72%	72%	71%	73%
	14/39 by 2026, 50% catchup Jan 2027	60%	67%	74%	76%	77%	78%	79%	80%
	14/39 by 2026, 80% catchup Jan 2027	60%	67%	74%	79%	79%	79%	80%	80%
	14/39 by 2026, 50% catchup Jan 2030	60%	67%	74%	71%	73%	75%	82%	83%
	14/39 by 2026, 80% catchup Jan 2030	60%	67%	74%	71%	73%	75%	86%	86%
Ecuador	Current Schedule: 2 doses	54%	52%	52%	52%	52%	52%	52%	52%
	14/39 by 2026	54%	52%	52%	50%	53%	56%	59%	62%
	14/39 by 2029	54%	52%	52%	52%	52%	52%	50%	53%
	14/39 by 2026, 50% catchup Jan 2027	54%	52%	52%	62%	62%	63%	63%	63%
	14/39 by 2026, 80% catchup Jan 2027	54%	52%	52%	69%	68%	66%	65%	63%
	14/39 by 2026, 50% catchup Jan 2030	54%	52%	52%	50%	53%	56%	72%	72%
	14/39 by 2026, 80% catchup Jan 2030	54%	52%	52%	50%	53%	56%	79%	77%
Grenada	Current Schedule: 2 doses	42%	52%	63%	74%	74%	74%	74%	74%
	14/39 by 2026	42%	52%	63%	74%	76%	77%	78%	79%
	14/39 by 2029	42%	52%	63%	74%	74%	74%	74%	76%
	14/39 by 2026, 50% catchup Jan 2027	42%	52%	63%	81%	81%	80%	80%	79%
	14/39 by 2026, 80% catchup Jan 2027	42%	52%	63%	86%	84%	83%	81%	80%
	14/39 by 2026, 50% catchup Jan 2030	42%	52%	63%	74%	76%	77%	84%	83%
	14/39 by 2026, 80% catchup Jan 2030	42%	52%	63%	74%	76%	77%	88%	86%
Guatemala	Current Schedule: 2 doses	63%	69%	69%	70%	70%	70%	70%	70%
	14/39 by 2026	63%	69%	69%	69%	71%	73%	75%	78%
	14/39 by 2029	63%	69%	69%	70%	70%	70%	69%	71%
	14/39 by 2026, 50% catchup Jan 2027	62%	69%	69%	75%	76%	77%	77%	78%
	14/39 by 2026, 80% catchup Jan 2027	62%	69%	69%	79%	79%	78%	78%	78%
	14/39 by 2026, 50% catchup Jan 2030	62%	69%	69%	69%	71%	73%	82%	82%
	14/39 by 2026, 80% catchup Jan 2030	62%	69%	69%	69%	71%	73%	85%	85%
Guyana	Current Schedule: 2 doses	70%	77%	78%	78%	78%	78%	78%	78%
	14/39 by 2026	70%	77%	78%	77%	79%	82%	84%	87%
	14/39 by 2029	70%	77%	78%	78%	78%	78%	77%	79%
	14/39 by 2026, 50% catchup Jan 2027	70%	77%	78%	79%	81%	83%	85%	87%
	14/39 by 2026, 80% catchup Jan 2027	70%	77%	78%	80%	82%	84%	85%	87%
	14/39 by 2026, 50% catchup Jan 2030	70%	77%	78%	77%	79%	82%	86%	88%
	14/39 by 2026, 80% catchup Jan 2030	70%	77%	78%	77%	79%	82%	88%	89%
Haiti	Current Schedule: 1 dose	18%	18%	18%	18%	18%	18%	18%	18%
	14/39 by 2026	18%	18%	18%	22%	30%	38%	46%	54%
	14/39 by 2029	18%	18%	18%	18%	18%	18%	22%	30%
	14/39 by 2026, 50% catchup Jan 2027	18%	18%	18%	55%	55%	55%	55%	55%
	14/39 by 2026, 80% catchup Jan 2027	18%	18%	18%	74%	70%	65%	60%	55%
	14/39 by 2026, 50% catchup Jan 2030	18%	18%	18%	22%	30%	38%	67%	67%
	14/39 by 2026, 80% catchup Jan 2030	18%	18%	18%	22%	30%	38%	79%	74%
Honduras	Current Schedule: 2 doses	66%	64%	63%	62%	62%	62%	62%	62%
	14/39 by 2026	66%	64%	63%	61%	62%	64%	66%	67%

	14/39 by 2029	66%	64%	63%	62%	62%	62%	61%	62%
	14/39 by 2026, 50% catchup Jan 2027	66%	64%	63%	71%	70%	69%	69%	68%
	14/39 by 2026, 80% catchup Jan 2027	66%	64%	63%	77%	74%	72%	70%	68%
	14/39 by 2026, 50% catchup Jan 2030	66%	64%	63%	61%	62%	64%	76%	75%
	14/39 by 2026, 80% catchup Jan 2030	66%	64%	63%	61%	62%	64%	82%	80%
Jamaica	Current Schedule: 2 doses	60%	73%	86%	86%	87%	87%	87%	87%
	14/39 by 2026	60%	73%	86%	86%	87%	87%	88%	88%
	14/39 by 2029	60%	73%	86%	86%	87%	87%	86%	87%
	14/39 by 2026, 50% catchup Jan 2027	60%	73%	86%	88%	88%	88%	88%	88%
	14/39 by 2026, 80% catchup Jan 2027	60%	73%	86%	88%	88%	88%	89%	89%
	14/39 by 2026, 50% catchup Jan 2030	60%	73%	86%	86%	87%	87%	89%	89%
	14/39 by 2026, 80% catchup Jan 2030	60%	73%	86%	86%	87%	87%	90%	90%
Nicaragua	Current Schedule: 2 doses	53%	60%	67%	75%	75%	75%	75%	75%
	14/39 by 2026	53%	60%	67%	74%	75%	78%	80%	82%
	14/39 by 2029	53%	60%	67%	75%	75%	75%	73%	75%
	14/39 by 2026, 50% catchup Jan 2027	53%	60%	67%	77%	78%	80%	81%	82%
	14/39 by 2026, 80% catchup Jan 2027	53%	60%	67%	79%	80%	81%	82%	82%
	14/39 by 2026, 50% catchup Jan 2030	53%	60%	67%	74%	75%	78%	84%	85%
	14/39 by 2026, 80% catchup Jan 2030	53%	60%	67%	74%	75%	78%	86%	87%
Saint Kitts and Nevis	Current Schedule: 2 doses	46%	57%	67%	77%	87%	87%	87%	87%
	14/39 by 2026	46%	57%	67%	76%	87%	87%	88%	88%
	14/39 by 2029	46%	57%	67%	77%	87%	87%	86%	87%
	14/39 by 2026, 50% catchup Jan 2027	46%	57%	67%	83%	88%	88%	88%	88%
	14/39 by 2026, 80% catchup Jan 2027	46%	57%	67%	87%	89%	89%	88%	88%
	14/39 by 2026, 50% catchup Jan 2030	46%	57%	67%	76%	87%	87%	89%	89%
	14/39 by 2026, 80% catchup Jan 2030	46%	57%	67%	76%	87%	87%	90%	90%
Saint Lucia	Current Schedule: 2 doses	43%	52%	61%	70%	80%	80%	80%	80%
	14/39 by 2026	43%	52%	61%	70%	79%	80%	80%	81%
	14/39 by 2029	43%	52%	61%	70%	80%	80%	79%	79%
	14/39 by 2026, 50% catchup Jan 2027	43%	52%	61%	80%	83%	82%	82%	81%
	14/39 by 2026, 80% catchup Jan 2027	43%	52%	61%	85%	86%	84%	83%	81%
	14/39 by 2026, 50% catchup Jan 2030	43%	52%	61%	70%	79%	80%	85%	84%
	14/39 by 2026, 80% catchup Jan 2030	43%	52%	61%	70%	79%	80%	88%	87%
Saint Vincent and The Grenadines	Current Schedule: 2 doses	80%	80%	80%	81%	80%	80%	80%	80%
	14/39 by 2026	80%	80%	80%	79%	81%	84%	86%	89%
	14/39 by 2029	80%	80%	80%	81%	80%	80%	79%	81%
	14/39 by 2026, 50% catchup Jan 2027	80%	80%	80%	80%	82%	84%	86%	89%
	14/39 by 2026, 80% catchup Jan 2027	80%	80%	80%	81%	83%	85%	87%	89%
	14/39 by 2026, 50% catchup Jan 2030	80%	80%	80%	79%	81%	84%	87%	90%
	14/39 by 2026, 80% catchup Jan 2030	80%	80%	80%	79%	81%	84%	88%	90%
Suriname	Current Schedule: 2 doses	38%	45%	53%	59%	66%	66%	66%	66%
	14/39 by 2026	38%	45%	53%	58%	66%	68%	69%	71%
	14/39 by 2029	38%	45%	53%	59%	66%	66%	65%	66%
	14/39 by 2026, 50% catchup Jan 2027	38%	45%	53%	70%	72%	72%	71%	71%
	14/39 by 2026, 80% catchup Jan 2027	38%	45%	53%	78%	76%	75%	73%	71%
	14/39 by 2026, 50% catchup Jan 2030	38%	45%	53%	58%	66%	68%	78%	78%
	14/39 by 2026, 80% catchup Jan 2030	38%	45%	53%	58%	66%	68%	83%	82%
Trinidad and Tobago	Current Schedule: 2 doses	71%	79%	79%	80%	80%	80%	80%	80%

	14/39 by 2026	71%	79%	79%	78%	81%	83%	86%	88%
	14/39 by 2029	71%	79%	79%	80%	80%	80%	78%	81%
	14/39 by 2026, 50% catchup Jan 2027	71%	79%	79%	80%	82%	84%	86%	88%
	14/39 by 2026, 80% catchup Jan 2027	71%	79%	79%	81%	83%	85%	87%	88%
	14/39 by 2026, 50% catchup Jan 2030	71%	79%	79%	78%	81%	83%	87%	89%
	14/39 by 2026, 80% catchup Jan 2030	71%	79%	79%	78%	81%	83%	88%	90%
Turks and Caicos	Current Schedule: 2 doses	81%	81%	82%	82%	82%	82%	82%	82%
	14/39 by 2026	81%	81%	82%	81%	83%	86%	89%	91%
	14/39 by 2029	81%	81%	82%	82%	82%	82%	81%	83%
	14/39 by 2026, 50% catchup Jan 2027	81%	81%	82%	81%	83%	86%	89%	91%
	14/39 by 2026, 80% catchup Jan 2027	81%	81%	82%	81%	84%	86%	89%	91%
	14/39 by 2026, 50% catchup Jan 2030	81%	81%	82%	81%	83%	86%	89%	91%
	14/39 by 2026, 80% catchup Jan 2030	81%	81%	82%	81%	83%	86%	89%	91%
Venezuela	Current Schedule: 2 doses	38%	40%	43%	42%	43%	43%	43%	43%
	14/39 by 2026	38%	40%	43%	42%	44%	46%	47%	49%
	14/39 by 2029	38%	40%	43%	42%	43%	43%	42%	44%
	14/39 by 2026, 50% catchup Jan 2027	38%	40%	43%	62%	59%	56%	53%	50%
	14/39 by 2026, 80% catchup Jan 2027	38%	40%	43%	74%	68%	62%	56%	51%
	14/39 by 2026, 50% catchup Jan 2030	38%	40%	43%	42%	44%	46%	66%	63%
	14/39 by 2026, 80% catchup Jan 2030	38%	40%	43%	42%	44%	46%	78%	72%

2.3 Eastern Mediterranean Region Countries

Table I: IPV-induced immunity projections under different schedules and scenarios, for Eastern Mediterranean region countries using one or two doses of IPV.

Country	Scenario	2024	2025	2026	2027	2028	2029	2030	2031
Afghanistan	Current Schedule: 2 doses	48%	51%	51%	52%	52%	52%	52%	52%
	14/39 by 2026	48%	51%	51%	52%	52%	52%	52%	52%
	14/39 by 2029	48%	51%	51%	52%	52%	52%	52%	52%
	14/39 by 2026, 50% catchup Jan 2027	48%	51%	51%	70%	65%	61%	57%	53%
	14/39 by 2026, 80% catchup Jan 2027	48%	51%	51%	80%	73%	67%	60%	54%
	14/39 by 2026, 50% catchup Jan 2030	48%	51%	51%	52%	52%	52%	70%	65%
	14/39 by 2026, 80% catchup Jan 2030	48%	51%	51%	52%	52%	52%	80%	73%
Djibouti	Current Schedule: 2 doses	45%	44%	45%	46%	47%	47%	47%	47%
	14/39 by 2026	45%	44%	45%	46%	51%	54%	57%	60%
	14/39 by 2029	45%	44%	45%	46%	47%	47%	48%	51%
	14/39 by 2026, 50% catchup Jan 2027	45%	44%	45%	65%	64%	62%	62%	61%
	14/39 by 2026, 80% catchup Jan 2027	45%	44%	45%	76%	72%	67%	64%	62%
	14/39 by 2026, 50% catchup Jan 2030	45%	44%	45%	46%	51%	54%	71%	71%
	14/39 by 2026, 80% catchup Jan 2030	45%	44%	45%	46%	51%	54%	80%	78%
Iran (Islamic Republic of)	Current Schedule: 2 doses	81%	85%	89%	89%	89%	89%	89%	89%
	14/39 by 2026	81%	85%	89%	88%	89%	89%	89%	90%
	14/39 by 2029	81%	85%	89%	89%	89%	89%	88%	89%
	14/39 by 2026, 50% catchup Jan 2027	81%	85%	89%	89%	89%	89%	90%	90%
	14/39 by 2026, 80% catchup Jan 2027	81%	85%	89%	89%	89%	90%	90%	90%
	14/39 by 2026, 50% catchup Jan 2030	81%	85%	89%	88%	89%	89%	90%	90%
	14/39 by 2026, 80% catchup Jan 2030	81%	85%	89%	88%	89%	89%	91%	91%
Iraq	Current Schedule: 2 doses	72%	71%	71%	71%	70%	70%	70%	70%

	14/39 by 2026	72%	71%	71%	70%	70%	70%	71%	71%
	14/39 by 2029	72%	71%	71%	71%	70%	70%	69%	70%
	14/39 by 2026, 50% catchup Jan 2027	72%	71%	71%	79%	77%	75%	74%	72%
	14/39 by 2026, 80% catchup Jan 2027	72%	71%	71%	84%	81%	78%	75%	72%
	14/39 by 2026, 50% catchup Jan 2030	72%	71%	71%	70%	70%	70%	80%	78%
	14/39 by 2026, 80% catchup Jan 2030	72%	71%	71%	70%	70%	70%	85%	82%
Lebanon	Current Schedule: 2 doses	39%	45%	51%	58%	64%	64%	64%	64%
	14/39 by 2026	39%	45%	51%	56%	64%	65%	66%	68%
	14/39 by 2029	39%	45%	51%	58%	64%	64%	63%	64%
	14/39 by 2026, 50% catchup Jan 2027	39%	45%	51%	69%	71%	70%	69%	68%
	14/39 by 2026, 80% catchup Jan 2027	39%	45%	51%	77%	75%	73%	71%	69%
	14/39 by 2026, 50% catchup Jan 2030	39%	45%	51%	56%	64%	65%	77%	76%
	14/39 by 2026, 80% catchup Jan 2030	39%	45%	51%	56%	64%	65%	83%	81%
Morocco	Current Schedule: 2 doses	66%	66%	67%	71%	76%	80%	85%	85%
	14/39 by 2026	66%	66%	67%	71%	76%	80%	84%	84%
	14/39 by 2029	66%	66%	67%	71%	76%	80%	84%	84%
	14/39 by 2026, 50% catchup Jan 2027	66%	66%	67%	81%	82%	84%	85%	85%
	14/39 by 2026, 80% catchup Jan 2027	66%	66%	67%	87%	86%	86%	86%	85%
	14/39 by 2026, 50% catchup Jan 2030	66%	66%	67%	71%	76%	80%	88%	87%
	14/39 by 2026, 80% catchup Jan 2030	66%	66%	67%	71%	76%	80%	89%	88%
Oman	Current Schedule: 2 doses	82%	82%	82%	82%	82%	82%	82%	82%
	14/39 by 2026	82%	82%	82%	80%	83%	85%	88%	90%
	14/39 by 2029	82%	82%	82%	82%	82%	82%	80%	83%
	14/39 by 2026, 50% catchup Jan 2027	82%	82%	82%	81%	83%	86%	88%	90%
	14/39 by 2026, 80% catchup Jan 2027	82%	82%	82%	81%	83%	86%	88%	90%
	14/39 by 2026, 50% catchup Jan 2030	82%	82%	82%	80%	83%	85%	88%	91%
	14/39 by 2026, 80% catchup Jan 2030	82%	82%	82%	80%	83%	85%	89%	91%
Pakistan	Current Schedule: 2 doses	67%	73%	79%	80%	80%	80%	80%	80%
	14/39 by 2026	67%	73%	79%	80%	80%	80%	80%	80%
	14/39 by 2029	67%	73%	79%	80%	80%	80%	80%	80%
	14/39 by 2026, 50% catchup Jan 2027	67%	73%	79%	85%	84%	83%	82%	81%
	14/39 by 2026, 80% catchup Jan 2027	67%	73%	79%	88%	86%	85%	83%	81%
	14/39 by 2026, 50% catchup Jan 2030	67%	73%	79%	80%	80%	80%	85%	84%
	14/39 by 2026, 80% catchup Jan 2030	67%	73%	79%	80%	80%	80%	88%	86%
Occupied Palestinian Territories	Current Schedule: 2 doses	46%	46%	45%	45%	44%	44%	44%	44%
	14/39 by 2026	46%	46%	45%	47%	55%	64%	73%	82%
	14/39 by 2029	46%	46%	45%	45%	44%	44%	47%	55%
	14/39 by 2026, 50% catchup Jan 2027	46%	46%	45%	48%	57%	65%	74%	82%
	14/39 by 2026, 80% catchup Jan 2027	46%	46%	45%	49%	57%	66%	74%	82%
	14/39 by 2026, 50% catchup Jan 2030	46%	46%	45%	47%	55%	64%	77%	85%
	14/39 by 2026, 80% catchup Jan 2030	46%	46%	45%	47%	55%	64%	79%	87%
Qatar	Current Schedule: 2 doses	80%	81%	81%	81%	81%	81%	81%	81%
	14/39 by 2026	80%	81%	81%	79%	82%	84%	87%	89%
	14/39 by 2029	80%	81%	81%	81%	81%	81%	79%	82%
	14/39 by 2026, 50% catchup Jan 2027	80%	81%	81%	80%	83%	85%	87%	90%
	14/39 by 2026, 80% catchup Jan 2027	80%	81%	81%	81%	83%	85%	87%	90%
	14/39 by 2026, 50% catchup Jan 2030	80%	81%	81%	79%	82%	84%	88%	90%
	14/39 by 2026, 80% catchup Jan 2030	80%	81%	81%	79%	82%	84%	88%	91%

Somalia	Current Schedule: 2 doses	32%	34%	36%	39%	38%	38%	38%	38%
	14/39 by 2026	32%	34%	36%	39%	38%	38%	38%	38%
	14/39 by 2029	32%	34%	36%	39%	38%	38%	38%	38%
	14/39 by 2026, 50% catchup Jan 2027	32%	34%	36%	62%	56%	51%	45%	40%
	14/39 by 2026, 80% catchup Jan 2027	32%	34%	36%	76%	67%	58%	49%	41%
	14/39 by 2026, 50% catchup Jan 2030	32%	34%	36%	39%	38%	38%	62%	56%
	14/39 by 2026, 80% catchup Jan 2030	32%	34%	36%	39%	38%	38%	76%	67%
Sudan	Current Schedule: 2 doses	51%	48%	45%	43%	44%	44%	44%	44%
	14/39 by 2026	51%	48%	45%	42%	44%	45%	47%	48%
	14/39 by 2029	51%	48%	45%	43%	44%	44%	43%	44%
	14/39 by 2026, 50% catchup Jan 2027	51%	48%	45%	60%	56%	54%	52%	49%
	14/39 by 2026, 80% catchup Jan 2027	51%	48%	45%	70%	63%	59%	54%	50%
	14/39 by 2026, 50% catchup Jan 2030	51%	48%	45%	42%	44%	45%	65%	63%
	14/39 by 2026, 80% catchup Jan 2030	51%	48%	45%	42%	44%	45%	76%	71%
Syrian Arab Republic	Current Schedule: 2 doses	56%	55%	55%	55%	55%	55%	55%	55%
	14/39 by 2026	56%	55%	55%	54%	56%	58%	60%	62%
	14/39 by 2029	56%	55%	55%	55%	55%	55%	54%	56%
	14/39 by 2026, 50% catchup Jan 2027	56%	55%	55%	67%	66%	65%	64%	63%
	14/39 by 2026, 80% catchup Jan 2027	56%	55%	55%	75%	72%	69%	66%	63%
	14/39 by 2026, 50% catchup Jan 2030	56%	55%	55%	54%	56%	58%	73%	72%
	14/39 by 2026, 80% catchup Jan 2030	56%	55%	55%	54%	56%	58%	81%	78%
Yemen	Current Schedule: 2 doses	43%	44%	46%	45%	44%	44%	44%	44%
	14/39 by 2026	43%	44%	46%	45%	44%	44%	44%	44%
	14/39 by 2029	43%	44%	46%	45%	44%	44%	44%	44%
	14/39 by 2026, 50% catchup Jan 2027	43%	44%	46%	65%	60%	55%	50%	45%
	14/39 by 2026, 80% catchup Jan 2027	43%	44%	46%	78%	70%	62%	54%	46%
	14/39 by 2026, 50% catchup Jan 2030	43%	44%	46%	45%	44%	44%	65%	60%
	14/39 by 2026, 80% catchup Jan 2030	43%	44%	46%	45%	44%	44%	77%	70%

2.4 European Region Countries

Table J: IPV-induced immunity projections under different schedules and scenarios, for European region countries using one or two doses of IPV.

Country	Scenario	2024	2025	2026	2027	2028	2029	2030	2031
Albania	Current Schedule: 2 doses	81%	80%	80%	80%	80%	80%	80%	80%
	14/39 by 2026	81%	80%	80%	79%	81%	84%	86%	89%
	14/39 by 2029	81%	80%	80%	80%	80%	80%	79%	81%
	14/39 by 2026, 50% catchup Jan 2027	81%	80%	80%	80%	82%	84%	87%	89%
	14/39 by 2026, 80% catchup Jan 2027	81%	80%	80%	81%	83%	85%	87%	89%
	14/39 by 2026, 50% catchup Jan 2030	81%	80%	80%	79%	81%	84%	87%	90%
	14/39 by 2026, 80% catchup Jan 2030	81%	80%	80%	79%	81%	84%	88%	90%
Azerbaijan	Current Schedule: 2 doses	80%	81%	82%	83%	83%	83%	83%	83%
	14/39 by 2026	80%	81%	82%	81%	82%	82%	83%	83%
	14/39 by 2029	80%	81%	82%	83%	83%	83%	82%	82%
	14/39 by 2026, 50% catchup Jan 2027	80%	81%	82%	85%	85%	84%	84%	83%
	14/39 by 2026, 80% catchup Jan 2027	80%	81%	82%	88%	86%	85%	84%	83%
	14/39 by 2026, 50% catchup Jan 2030	80%	81%	82%	81%	82%	82%	86%	86%

	14/39 by 2026, 80% catchup Jan 2030	80%	81%	82%	81%	82%	82%	88%	87%
Kyrgyzstan	Current Schedule: 2 doses	67%	71%	77%	81%	82%	82%	82%	82%
	14/39 by 2026	67%	71%	77%	81%	82%	82%	82%	82%
	14/39 by 2029	67%	71%	77%	81%	82%	82%	82%	82%
	14/39 by 2026, 50% catchup Jan 2027	67%	71%	77%	86%	85%	84%	83%	82%
	14/39 by 2026, 80% catchup Jan 2027	67%	71%	77%	88%	87%	85%	84%	82%
	14/39 by 2026, 50% catchup Jan 2030	67%	71%	77%	81%	82%	82%	86%	85%
	14/39 by 2026, 80% catchup Jan 2030	67%	71%	77%	81%	82%	82%	89%	87%
Republic of Moldova	Current Schedule: 2 doses	74%	75%	76%	77%	77%	77%	77%	77%
	14/39 by 2026	74%	75%	76%	79%	81%	81%	81%	81%
	14/39 by 2029	74%	75%	76%	77%	77%	77%	79%	81%
	14/39 by 2026, 50% catchup Jan 2027	74%	75%	76%	83%	83%	82%	81%	81%
	14/39 by 2026, 80% catchup Jan 2027	74%	75%	76%	85%	85%	83%	81%	81%
	14/39 by 2026, 50% catchup Jan 2030	74%	75%	76%	79%	81%	81%	86%	85%
	14/39 by 2026, 80% catchup Jan 2030	74%	75%	76%	79%	81%	81%	88%	87%
Tajikistan	Current Schedule: 2 doses	75%	79%	84%	88%	90%	90%	90%	90%
	14/39 by 2026	75%	79%	84%	88%	90%	90%	90%	90%
	14/39 by 2029	75%	79%	84%	88%	90%	90%	90%	90%
	14/39 by 2026, 50% catchup Jan 2027	75%	79%	84%	90%	90%	90%	90%	90%
	14/39 by 2026, 80% catchup Jan 2027	75%	79%	84%	91%	91%	91%	90%	90%
	14/39 by 2026, 50% catchup Jan 2030	75%	79%	84%	88%	90%	90%	91%	90%
	14/39 by 2026, 80% catchup Jan 2030	75%	79%	84%	88%	90%	90%	91%	91%
Ukraine	Current Schedule: 2 doses	66%	64%	63%	63%	63%	63%	63%	63%
	14/39 by 2026	66%	64%	63%	62%	65%	67%	70%	72%
	14/39 by 2029	66%	64%	63%	63%	63%	63%	63%	65%
	14/39 by 2026, 50% catchup Jan 2027	66%	64%	63%	72%	72%	73%	73%	73%
	14/39 by 2026, 80% catchup Jan 2027	66%	64%	63%	78%	77%	76%	74%	73%
	14/39 by 2026, 50% catchup Jan 2030	66%	64%	63%	62%	65%	67%	79%	79%
	14/39 by 2026, 80% catchup Jan 2030	66%	64%	63%	62%	65%	67%	84%	83%
Uzbekistan	Current Schedule: 2 doses	75%	79%	84%	88%	88%	88%	88%	88%
	14/39 by 2026	75%	79%	84%	88%	88%	88%	88%	88%
	14/39 by 2029	75%	79%	84%	88%	88%	88%	88%	88%
	14/39 by 2026, 50% catchup Jan 2027	75%	79%	84%	89%	89%	89%	88%	88%
	14/39 by 2026, 80% catchup Jan 2027	75%	79%	84%	90%	90%	89%	89%	88%
	14/39 by 2026, 50% catchup Jan 2030	75%	79%	84%	88%	88%	88%	90%	89%
	14/39 by 2026, 80% catchup Jan 2030	75%	79%	84%	88%	88%	88%	90%	90%

2.5 South East Asia Region Countries

Table K: IPV-induced immunity projections under different schedules and scenarios, for South East Asia region countries using one or two doses of IPV.

Country	Scenario	2024	2025	2026	2027	2028	2029	2030	2031
Bangladesh	Current Schedule: 2 doses	69%	69%	69%	69%	69%	69%	69%	69%
	14/39 by 2026	69%	69%	69%	66%	71%	76%	81%	86%
	14/39 by 2029	69%	69%	69%	69%	69%	69%	66%	71%
	14/39 by 2026, 50% catchup Jan 2027	69%	69%	69%	66%	71%	76%	81%	86%
	14/39 by 2026, 80% catchup Jan 2027	69%	69%	69%	67%	72%	76%	81%	86%

	14/39 by 2026, 50% catchup Jan 2030	69%	69%	69%	66%	71%	76%	82%	87%
	14/39 by 2026, 80% catchup Jan 2030	69%	69%	69%	66%	71%	76%	82%	87%
Bhutan	Current Schedule: 2 doses	74%	80%	86%	89%	89%	89%	89%	89%
	14/39 by 2026	74%	80%	86%	88%	88%	88%	88%	88%
	14/39 by 2029	74%	80%	86%	89%	89%	89%	88%	88%
	14/39 by 2026, 50% catchup Jan 2027	74%	80%	86%	89%	89%	89%	89%	88%
	14/39 by 2026, 80% catchup Jan 2027	74%	80%	86%	90%	90%	89%	89%	88%
	14/39 by 2026, 50% catchup Jan 2030	74%	80%	86%	88%	88%	88%	89%	89%
	14/39 by 2026, 80% catchup Jan 2030	74%	80%	86%	88%	88%	88%	90%	90%
Indonesia	Current Schedule: 2 doses	59%	63%	68%	72%	74%	74%	74%	74%
	14/39 by 2026	59%	63%	68%	72%	75%	75%	75%	75%
	14/39 by 2029	59%	63%	68%	72%	74%	74%	75%	75%
	14/39 by 2026, 50% catchup Jan 2027	59%	63%	68%	81%	80%	79%	77%	76%
	14/39 by 2026, 80% catchup Jan 2027	59%	63%	68%	86%	84%	81%	79%	76%
	14/39 by 2026, 50% catchup Jan 2030	59%	63%	68%	72%	75%	75%	82%	81%
	14/39 by 2026, 80% catchup Jan 2030	59%	63%	68%	72%	75%	75%	87%	84%
Maldives	Current Schedule: 1 dose	81%	81%	82%	82%	82%	82%	82%	82%
	14/39 by 2026	81%	81%	82%	84%	86%	87%	89%	90%
	14/39 by 2029	81%	81%	82%	82%	82%	82%	84%	86%
	14/39 by 2026, 50% catchup Jan 2027	81%	81%	82%	88%	88%	89%	90%	90%
	14/39 by 2026, 80% catchup Jan 2027	81%	81%	82%	90%	90%	90%	90%	90%
	14/39 by 2026, 50% catchup Jan 2030	81%	81%	82%	84%	86%	87%	90%	91%
	14/39 by 2026, 80% catchup Jan 2030	81%	81%	82%	84%	86%	87%	91%	91%
Myanmar	Current Schedule: 1 dose	49%	48%	48%	51%	50%	50%	50%	50%
	14/39 by 2026	49%	48%	48%	51%	54%	57%	60%	63%
	14/39 by 2029	49%	48%	48%	51%	50%	50%	51%	54%
	14/39 by 2026, 50% catchup Jan 2027	49%	48%	48%	70%	68%	66%	65%	63%
	14/39 by 2026, 80% catchup Jan 2027	49%	48%	48%	81%	76%	72%	68%	64%
	14/39 by 2026, 50% catchup Jan 2030	49%	48%	48%	51%	54%	57%	74%	72%
	14/39 by 2026, 80% catchup Jan 2030	49%	48%	48%	51%	54%	57%	82%	78%
Nepal	Current Schedule: 2 doses	60%	65%	70%	74%	79%	79%	79%	79%
	14/39 by 2026	60%	65%	70%	74%	79%	79%	79%	79%
	14/39 by 2029	60%	65%	70%	74%	79%	79%	79%	79%
	14/39 by 2026, 50% catchup Jan 2027	60%	65%	70%	79%	82%	81%	80%	79%
	14/39 by 2026, 80% catchup Jan 2027	60%	65%	70%	81%	84%	83%	81%	79%
	14/39 by 2026, 50% catchup Jan 2030	60%	65%	70%	74%	79%	79%	83%	82%
	14/39 by 2026, 80% catchup Jan 2030	60%	65%	70%	74%	79%	79%	86%	84%
Sri Lanka	Current Schedule: 2 doses	72%	72%	72%	72%	72%	72%	72%	72%
	14/39 by 2026	72%	72%	72%	69%	74%	78%	83%	87%
	14/39 by 2029	72%	72%	72%	72%	72%	72%	69%	74%
	14/39 by 2026, 50% catchup Jan 2027	72%	72%	72%	70%	74%	79%	83%	87%
	14/39 by 2026, 80% catchup Jan 2027	72%	72%	72%	70%	75%	79%	83%	87%
	14/39 by 2026, 50% catchup Jan 2030	72%	72%	72%	69%	74%	78%	83%	87%
	14/39 by 2026, 80% catchup Jan 2030	72%	72%	72%	69%	74%	78%	84%	88%
Thailand	Current Schedule: 2 doses	71%	72%	73%	75%	77%	77%	77%	77%
	14/39 by 2026	71%	72%	73%	74%	77%	80%	82%	84%
	14/39 by 2029	71%	72%	73%	75%	77%	77%	76%	78%
	14/39 by 2026, 50% catchup Jan 2027	71%	72%	73%	79%	80%	82%	83%	85%

	14/39 by 2026, 80% catchup Jan 2027	71%	72%	73%	82%	82%	82%	84%	85%
	14/39 by 2026, 50% catchup Jan 2030	71%	72%	73%	74%	77%	80%	85%	87%
	14/39 by 2026, 80% catchup Jan 2030	71%	72%	73%	74%	77%	80%	87%	88%
Timor-Leste	Current Schedule: 1 dose	53%	53%	53%	53%	53%	53%	53%	53%
	14/39 by 2026	53%	53%	53%	54%	59%	64%	69%	74%
	14/39 by 2029	53%	53%	53%	53%	53%	53%	54%	59%
	14/39 by 2026, 50% catchup Jan 2027	53%	53%	53%	72%	72%	73%	74%	74%
	14/39 by 2026, 80% catchup Jan 2027	53%	53%	53%	82%	80%	78%	76%	74%
	14/39 by 2026, 50% catchup Jan 2030	53%	53%	53%	54%	59%	64%	79%	80%
	14/39 by 2026, 80% catchup Jan 2030	53%	53%	53%	54%	59%	64%	85%	83%

2.6 Western Pacific Region Countries

Table L: IPV-induced immunity projections under different schedules and scenarios, for Western Pacific region countries using one or two doses of IPV.

Country	Scenario	2024	2025	2026	2027	2028	2029	2030	2031
Cambodia	Current Schedule: 1 dose	57%	56%	56%	56%	56%	56%	56%	56%
	14/39 by 2026	57%	56%	56%	57%	62%	67%	72%	77%
	14/39 by 2029	57%	56%	56%	56%	56%	56%	57%	62%
	14/39 by 2026, 50% catchup Jan 2027	57%	56%	56%	73%	74%	75%	76%	77%
	14/39 by 2026, 80% catchup Jan 2027	57%	56%	56%	83%	82%	80%	79%	78%
	14/39 by 2026, 50% catchup Jan 2030	57%	56%	56%	57%	62%	67%	81%	82%
	14/39 by 2026, 80% catchup Jan 2030	57%	56%	56%	57%	62%	67%	86%	85%
China	Current Schedule: 2 doses	67%	67%	67%	67%	67%	67%	67%	67%
	14/39 by 2026	67%	67%	67%	67%	73%	78%	84%	90%
	14/39 by 2029	67%	67%	67%	67%	67%	67%	67%	73%
	14/39 by 2026, 50% catchup Jan 2027	67%	67%	67%	68%	73%	79%	84%	90%
	14/39 by 2026, 80% catchup Jan 2027	67%	67%	67%	68%	74%	79%	84%	90%
	14/39 by 2026, 50% catchup Jan 2030	67%	67%	67%	67%	73%	78%	85%	90%
	14/39 by 2026, 80% catchup Jan 2030	67%	67%	67%	67%	73%	78%	85%	91%
Cook Islands	Current Schedule: 1 dose	74%	73%	73%	74%	75%	75%	75%	75%
	14/39 by 2026	74%	73%	73%	75%	77%	78%	80%	81%
	14/39 by 2029	74%	73%	73%	74%	75%	75%	76%	77%
	14/39 by 2026, 50% catchup Jan 2027	74%	73%	73%	83%	83%	82%	82%	81%
	14/39 by 2026, 80% catchup Jan 2027	74%	73%	73%	87%	86%	85%	83%	81%
	14/39 by 2026, 50% catchup Jan 2030	74%	73%	73%	75%	77%	78%	85%	85%
	14/39 by 2026, 80% catchup Jan 2030	74%	73%	73%	75%	77%	78%	89%	87%
Fiji	Current Schedule: 2 doses	64%	64%	66%	72%	78%	84%	88%	88%
	14/39 by 2026	64%	64%	66%	73%	79%	85%	90%	90%
	14/39 by 2029	64%	64%	66%	72%	78%	84%	90%	90%
	14/39 by 2026, 50% catchup Jan 2027	64%	64%	66%	82%	85%	88%	90%	90%
	14/39 by 2026, 80% catchup Jan 2027	64%	64%	66%	88%	89%	90%	90%	90%
	14/39 by 2026, 50% catchup Jan 2030	64%	64%	66%	73%	79%	85%	91%	90%
	14/39 by 2026, 80% catchup Jan 2030	64%	64%	66%	73%	79%	85%	91%	91%
Kiribati	Current Schedule: 2 doses	61%	61%	62%	68%	74%	79%	84%	84%
	14/39 by 2026	61%	61%	62%	69%	75%	81%	85%	85%
	14/39 by 2029	61%	61%	62%	68%	74%	79%	85%	85%

	14/39 by 2026, 50% catchup Jan 2027	61%	61%	62%	80%	82%	84%	86%	85%
	14/39 by 2026, 80% catchup Jan 2027	61%	61%	62%	86%	87%	87%	86%	85%
	14/39 by 2026, 50% catchup Jan 2030	61%	61%	62%	69%	75%	81%	88%	87%
	14/39 by 2026, 80% catchup Jan 2030	61%	61%	62%	69%	75%	81%	90%	89%
Lao People's Democratic Republic	Current Schedule: 2 doses	51%	52%	54%	59%	64%	67%	70%	70%
	14/39 by 2026	51%	52%	54%	60%	65%	69%	72%	72%
	14/39 by 2029	51%	52%	54%	59%	64%	67%	71%	71%
	14/39 by 2026, 50% catchup Jan 2027	51%	52%	54%	75%	75%	75%	75%	73%
	14/39 by 2026, 80% catchup Jan 2027	51%	52%	54%	84%	81%	79%	76%	73%
	14/39 by 2026, 50% catchup Jan 2030	51%	52%	54%	60%	65%	69%	81%	79%
	14/39 by 2026, 80% catchup Jan 2030	51%	52%	54%	60%	65%	69%	86%	83%
Mongolia	Current Schedule: 1 dose	78%	78%	78%	79%	79%	79%	79%	79%
	14/39 by 2026	78%	78%	78%	80%	82%	84%	87%	89%
	14/39 by 2029	78%	78%	78%	79%	79%	79%	80%	82%
	14/39 by 2026, 50% catchup Jan 2027	78%	78%	78%	85%	86%	87%	88%	89%
	14/39 by 2026, 80% catchup Jan 2027	78%	78%	78%	89%	89%	89%	89%	89%
	14/39 by 2026, 50% catchup Jan 2030	78%	78%	78%	80%	82%	84%	89%	90%
	14/39 by 2026, 80% catchup Jan 2030	78%	78%	78%	80%	82%	84%	90%	90%
Nauru	Current Schedule: 1 dose	64%	64%	64%	64%	64%	64%	64%	64%
	14/39 by 2026	64%	64%	64%	65%	71%	78%	84%	90%
	14/39 by 2029	64%	64%	64%	64%	64%	64%	65%	71%
	14/39 by 2026, 50% catchup Jan 2027	64%	64%	64%	78%	81%	84%	87%	90%
	14/39 by 2026, 80% catchup Jan 2027	64%	64%	64%	86%	87%	88%	89%	90%
	14/39 by 2026, 50% catchup Jan 2030	64%	64%	64%	65%	71%	78%	87%	90%
	14/39 by 2026, 80% catchup Jan 2030	64%	64%	64%	65%	71%	78%	90%	91%
Papua New Guinea	Current Schedule: 2 doses	27%	30%	32%	34%	34%	34%	34%	34%
	14/39 by 2026	27%	30%	32%	34%	34%	34%	34%	34%
	14/39 by 2029	27%	30%	32%	34%	34%	34%	34%	34%
	14/39 by 2026, 50% catchup Jan 2027	27%	30%	32%	60%	54%	48%	42%	36%
	14/39 by 2026, 80% catchup Jan 2027	27%	30%	32%	75%	65%	56%	46%	37%
	14/39 by 2026, 50% catchup Jan 2030	27%	30%	32%	34%	34%	34%	60%	54%
	14/39 by 2026, 80% catchup Jan 2030	27%	30%	32%	34%	34%	34%	75%	65%
Philippines	Current Schedule: 2 doses	58%	63%	68%	71%	72%	72%	72%	72%
	14/39 by 2026	58%	63%	68%	71%	72%	72%	72%	72%
	14/39 by 2029	58%	63%	68%	71%	72%	72%	72%	72%
	14/39 by 2026, 50% catchup Jan 2027	58%	63%	68%	80%	79%	77%	74%	72%
	14/39 by 2026, 80% catchup Jan 2027	58%	63%	68%	86%	83%	79%	76%	73%
	14/39 by 2026, 50% catchup Jan 2030	58%	63%	68%	71%	72%	72%	81%	79%
	14/39 by 2026, 80% catchup Jan 2030	58%	63%	68%	71%	72%	72%	86%	83%
Samoa	Current Schedule: 1 dose	58%	59%	59%	60%	61%	61%	61%	61%
	14/39 by 2026	58%	59%	59%	61%	66%	70%	74%	78%
	14/39 by 2029	58%	59%	59%	60%	61%	61%	62%	66%
	14/39 by 2026, 50% catchup Jan 2027	58%	59%	59%	76%	77%	77%	78%	78%
	14/39 by 2026, 80% catchup Jan 2027	58%	59%	59%	85%	83%	82%	80%	78%
	14/39 by 2026, 50% catchup Jan 2030	58%	59%	59%	61%	66%	70%	82%	83%
	14/39 by 2026, 80% catchup Jan 2030	58%	59%	59%	61%	66%	70%	87%	86%
Solomon Islands	Current Schedule: 1 dose	56%	55%	54%	53%	53%	53%	53%	53%
	14/39 by 2026	56%	55%	54%	55%	60%	65%	70%	76%

	14/39 by 2029	56%	55%	54%	53%	53%	53%	54%	60%
	14/39 by 2026, 50% catchup Jan 2027	56%	55%	54%	72%	73%	74%	75%	76%
	14/39 by 2026, 80% catchup Jan 2027	56%	55%	54%	82%	81%	79%	78%	76%
	14/39 by 2026, 50% catchup Jan 2030	56%	55%	54%	55%	60%	65%	80%	81%
	14/39 by 2026, 80% catchup Jan 2030	56%	55%	54%	55%	60%	65%	86%	84%
Tonga	Current Schedule: 1 dose	64%	64%	64%	64%	64%	64%	64%	64%
	14/39 by 2026	64%	64%	64%	66%	72%	78%	84%	89%
	14/39 by 2029	64%	64%	64%	64%	64%	64%	66%	72%
	14/39 by 2026, 50% catchup Jan 2027	64%	64%	64%	78%	81%	84%	87%	89%
	14/39 by 2026, 80% catchup Jan 2027	64%	64%	64%	86%	87%	88%	89%	90%
	14/39 by 2026, 50% catchup Jan 2030	64%	64%	64%	66%	72%	78%	87%	90%
	14/39 by 2026, 80% catchup Jan 2030	64%	64%	64%	66%	72%	78%	90%	91%
Vanuatu	Current Schedule: 1 dose	48%	47%	47%	49%	50%	50%	50%	50%
	14/39 by 2026	48%	47%	47%	50%	55%	59%	62%	66%
	14/39 by 2029	48%	47%	47%	49%	50%	50%	51%	55%
	14/39 by 2026, 50% catchup Jan 2027	48%	47%	47%	69%	69%	68%	68%	67%
	14/39 by 2026, 80% catchup Jan 2027	48%	47%	47%	81%	78%	74%	71%	67%
	14/39 by 2026, 50% catchup Jan 2030	48%	47%	47%	50%	55%	59%	76%	75%
	14/39 by 2026, 80% catchup Jan 2030	48%	47%	47%	50%	55%	59%	84%	80%
Viet Nam	Current Schedule: 2 doses	71%	72%	73%	75%	76%	76%	76%	76%
	14/39 by 2026	71%	72%	73%	75%	77%	77%	77%	77%
	14/39 by 2029	71%	72%	73%	75%	76%	76%	77%	77%
	14/39 by 2026, 50% catchup Jan 2027	71%	72%	73%	83%	82%	80%	79%	78%
	14/39 by 2026, 80% catchup Jan 2027	71%	72%	73%	87%	85%	82%	80%	78%
	14/39 by 2026, 50% catchup Jan 2030	71%	72%	73%	75%	77%	77%	83%	82%
	14/39 by 2026, 80% catchup Jan 2030	71%	72%	73%	75%	77%	77%	87%	85%

3 Gains from switching to 14, 39 week schedule

3.1 African Region countries

3.1.1 Single dose countries

Table M: Possible immunity gains which could be achieved from switching from the current single-dose schedule to the 14/39 week schedule, African region countries.

Country	Age at first dose (weeks)	2030	2030: 14/39	Possible Gains
Burundi	14	57%	81%	+23%
Kenya	14	59%	81%	+22%
Mauritania	14	61%	80%	+19%
Liberia	14	57%	76%	+19%
Guinea-Bissau	14	50%	69%	+19%
Benin	14	49%	62%	+13%
Gabon	14	47%	60%	+13%
Equatorial Guinea	14	50%	62%	+12%
Guinea	14	36%	47%	+11%
Central African Republic	14	31%	41%	+10%

3.1.2 Two-dose countries

Table N: Possible immunity gains which could be achieved from switching from the current two-dose schedule to the 14/39 week schedule, African region countries.

Country	Age at first dose (weeks)	Age at second dose (weeks)	2030	2030: 14/39	Possible Gains
Uganda	6	14	67%	81%	+14%
Senegal	6	14	64%	75%	+12%
Cote d'Ivoire	6	14	54%	63%	+9%
Nigeria	6	14	44%	52%	+8%

3.2 Americas Region Countries

3.2.1 Single dose countries

Table O: Possible immunity gains which could be achieved from switching from the current single-dose schedule to the 14/39 week schedule, Americas region countries.

Country	Age at first dose	2030	2030: 14/39	Possible Gains
Haiti	6	18%	54%	+36%

3.2.2 Two-dose countries

Table P: Possible immunity gains which could be achieved from switching from the current two-dose schedule to the 14/39 week schedule, Americas region countries.

Country	Age at first dose (weeks)	Age at second dose (weeks)	2030	2030: 14/39	Possible Gains
Ecuador	9	17	52%	62%	+10%
Antigua and Barbuda	9	17	75%	85%	+10%
Guyana	9	17	78%	87%	+9%
Turks and Caicos	9	17	82%	91%	+9%
Trinidad and Tobago	9	17	80%	88%	+9%
Saint Vincent and The Grenadines	9	17	80%	89%	+8%
Montserrat	9	17	73%	81%	+8%
Guatemala	9	17	70%	78%	+7%
Barbados	9	17	73%	80%	+7%
Nicaragua	9	17	75%	82%	+7%
Dominica	9	17	72%	79%	+7%
Anguilla	9	17	79%	86%	+7%
Belize	9	17	72%	78%	+6%
Bahamas	9	17	69%	75%	+6%
Venezuela	9	17	43%	49%	+6%
Honduras	9	17	62%	67%	+5%
Grenada	6	26	74%	79%	+5%
Suriname	9	17	66%	71%	+5%
Jamaica	6	26	87%	88%	+2%
Saint Lucia	9	26	80%	81%	+1%
Saint Kitts and Nevis	9	26	87%	88%	+1%

3.3 Eastern Mediterranean countries

3.3.1 Two-dose countries

Table Q: Possible immunity gains which could be achieved from switching from the current two-dose schedule to the 14/39 week schedule, Eastern Mediterranean region countries.

Country	Age at first dose (weeks)	Age at second dose (weeks)	2030	2030: 14/39	Possible Gains
Occupied Palestinian Territories	4	9	44%	82%	+38%
Djibouti	6	14	47%	60%	+13%
Oman	9	17	82%	90%	+9%
Qatar	9	17	81%	89%	+9%
Syrian Arab Republic	8	16	55%	62%	+7%
Sudan	6	14	44%	48%	+4%
Lebanon	9	17	64%	68%	+4%
Iraq	17	26	70%	71%	+2%
Iran (Islamic Republic of)	17	26	89%	90%	+1%

3.4 European Region Countries

3.4.1 Two-dose countries

Table R: Possible immunity gains which could be achieved from switching from the current two-dose schedule to the 14/39 week schedule, European region countries.

Country	Age at first dose (weeks)	Age at second dose (weeks)	2030	2030: 14/39	Possible Gains
Ukraine	9	17	63%	72%	+9%
Albania	9	17	80%	89%	+8%
Republic of Moldova	26	104	77%	81%	+4%

3.5 South East Asia Region countries

3.5.1 One dose countries

Table S: Possible immunity gains which could be achieved from switching from the current single-dose schedule to the 14/39 week schedule, South East Asia region countries.

Country	Age at first dose (weeks)	2030	2030: 14/39	Possible Gains
Timor-Leste	14	53%	74%	+20%
Myanmar	17	50%	63%	+12%
Maldives	26	82%	90%	+9%

3.5.2 Two-dose countries

Table T: Possible immunity gains which could be achieved from switching from the current two-dose schedule to the 14/39 week schedule, South East Asia region countries.

Country	Age at first dose (weeks)	Age at second dose (weeks)	2030	2030: 14/39	Possible Gains
Bangladesh	6	14	69%	86%	+17%
Sri Lanka	9	17	72%	87%	+15%
Thailand	9	17	77%	84%	+7%
Indonesia	17	39	74%	75%	+1%

3.6 Western Pacific Region Countries

3.6.1 Single dose countries

Table U: Possible immunity gains which could be achieved from switching from the current single-dose schedule to the 14/39 week schedule, Western Pacific region countries.

Country	Age at first dose (weeks)	2030	2030: 14/39	Possible Gains
Nauru	14	64%	90%	+26%
Tonga	14	64%	89%	+25%
Solomon Islands	14	53%	76%	+23%
Cambodia	14	56%	77%	+21%
Samoa	14	61%	78%	+17%
Vanuatu	14	50%	66%	+17%
Mongolia	22	79%	89%	+10%
Cook Islands	22	75%	81%	+6%

3.6.2 Two-dose countries

Table V: Possible immunity gains which could be achieved from switching from the current two-dose schedule to the 14/39 week schedule, Western Pacific region countries.

Country	Age at first dose (weeks)	Age at second dose (weeks)	2030	2030: 14/39	Possible Gains
China	9	13	67%	90%	+23%
Lao People's Democratic Republic	14	52	70%	72%	+3%
Fiji	14	52	88%	90%	+2%
Kiribati	14	52	84%	85%	+1%
Viet Nam	22	39	76%	77%	+1%

3.7 Equilibrium immunity estimates: accounting for delay

Table W: The estimated 'equilibrium immunity' (i.e. more than five years after schedule change) on the early and recommended schedules, accounting for delay, where delay data is available

Country	Age at first dose (weeks)	Age at second dose (weeks)	Current Schedule	6/14 week schedule	14/39 week schedule
Angola	17	39	47%	46%	48%
Benin	14		54%	63%	63%
Burundi	14		64%	71%	79%
Chad	14	39	64%	67%	64%
Democratic Republic of the Congo	14	39	53%	55%	53%
Gabon	14		52%	59%	63%
Gambia	17	39	69%	60%	70%
Ghana	14	39	84%	78%	84%
Guinea	14		40%	47%	47%
Kenya	14		63%	71%	81%
Lesotho	14	39	76%	70%	76%
Liberia	14		65%	73%	77%
Madagascar	14	39	57%	55%	57%
Malawi	14	39	80%	75%	80%
Mali	14	39	64%	62%	64%
Mauritania	14		69%	77%	82%
Namibia	14	39	74%	66%	74%
Nigeria	6	14	49%	49%	52%
Rwanda	14	39	86%	75%	86%
Senegal	6	14	72%	72%	74%
Sierra Leone	14	39	79%	73%	79%
Togo	14	39	75%	72%	75%
Uganda	6	14	75%	75%	77%
United Republic of Tanzania	14	39	83%	78%	83%
Zimbabwe	14	39	80%	71%	80%
Guatemala	9	17	72%	67%	77%
Haiti	6		31%	55%	54%
Pakistan	14	39	80%	75%	80%
Yemen	14	39	42%	41%	42%
Tajikistan	13	39	89%	76%	89%
Bangladesh	6	14	81%	81%	85%
Indonesia	17	39	71%	66%	72%
Maldives	26		82%	74%	90%
Myanmar	17		52%	54%	62%
Timor-Leste	14		59%	67%	73%
Philippines	14	39	74%	71%	74%

4 Methods

4.1 Coverage decline model

To fit the model which describes how coverage declines with age of scheduled administration, for each country we take the WUENIC (WHO/UNICEF estimates of national immunization coverage) coverage estimates, matched to the ages in the published routine immunisation schedule for that country. Coverage estimates for BCG, IPV first dose, IPV second dose (2023 only), Yellow Fever, Rotavirus, Hepatitis B birth dose, Measles-containing vaccine first and second doses, and DTP (Diphtheria, Tetanus, Pertussis) first and third doses, as well as *Haemophilus influenzae* type b third dose, were used World Health Organisation. These WUENIC estimates are matched to the ages of administration from official published schedules World Health Organisation. For multi-dose vaccines, it is assumed that the coverage for two or three doses represents the typical coverage of a vaccine given at the ages of administration of the second or third doses. WUENIC data are available for 109 of the 114 one or two-dose countries or territories. For the remaining five, sufficient administrative coverage data are available for Anguilla, Turks and Caicos, and the British Virgin Islands (all two doses). Administrative data is available for Montserrat (two doses) from 2021 onwards, thus estimates are provided from 2027 onwards, but these are not included in wider summary statistics. Curaçao (one dose) is excluded from the analyses.

For the coverage $c(w)$ of a particular country at week w we fit a linear model to the logit of the coverage:

$$\log\left(\frac{c(w)}{1-c(w)}\right) = \beta_0 + \beta_1 w + \epsilon, \tag{1}$$

where

$$\epsilon \sim N(0, \sigma^2), \tag{2}$$

where σ^2 is the variance parameter to be estimated.

4.2 Seroconversion with age model

A review of type-2 seroconversion by age of IPV dose administration for one or two full or fractional doses of IPV Grassly (2014) was updated. A total of nineteen studies, some with multiple arms, conducted between 1985 and 2022 were considered: Zaman et al. (2021), Asturias et al. (2016),Saleem et al. (2021), Ahmad et al. (2022), Aziz et al. (2022), Tagbo et al. (2022), Snider et al. (2019), Resik et al. (2020), Linder et al. (1995), McBean et al. (1988), Mohammed et al. (2010), Resik et al. (2010), Resik et al. (2013), Simasathien et al. (1994), Anand et al. (2015), Group (2007), Simoes et al. (1985), Bandyopadhyay et al. (2021), Nirmal et al. (1998), details of which are shown in Table X. The total numbers of participants were 3816, 2211, 1202 and 1163 for single full doses, two full doses, single fractional doses, and two fractional doses, respectively. Doses were given at a range of ages, from one to 48 weeks of age. We estimated the overall association of type-2 seroconversion as a function of age of administration through fitting a binomial regression model to these study estimates. Age at first dose (weeks), and the interval between doses were used as potential predictors as well as whether studies were conducted per or post the 2016 switch from usage of trivalent oral polio vaccine to bOPV in RI .

Table X: Studies used in seroconversion model. N= number of participants, n= number of participants who had seroconverted, Switch refers to whether the stuy was carried out before or after the 2016 switch from tOPV to bOPV.

**Study**	**Age dose 1 (weeks)**	**Age at dose 2 (weeks)**	**Switch**	**n**	**N**	**Type**
Zaman et al. (2021)	14	-	post	204	305	Single Full dose
Zaman et al. (2021)	6	-	post	77	321	Single Full dose
Asturias et al. (2016)	14	-	pre	147	184	Single Full dose
Asturias et al. (2016)	14	-	pre	147	196	Single Full dose
Saleem et al. (2021)	14	-	post	85	125	Single Full dose
Ahmad et al. (2022)	14	-	post	110	162	Single Full dose
Aziz et al. (2022)	40	-	post	129	137	Single Full dose
Tagbo et al. (2022)	14	-	post	193	268	Single Full dose
Snider et al. (2019)	6	-	post	70	268	Single Full dose
Snider et al. (2019)	14	-	post	123	267	Single Full dose
Snider et al. (2019)	14	-	post	126	271	Single Full dose
Resik et al. (2020)	16	-	post	19	25	Single Full dose
Linder et al. (1995)	1	-	pre	6	39	Single Full dose
McBean et al. (1988)	8	-	pre	109	311	Single Full dose
McBean et al. (1988)	8	-	pre	139	324	Single Full dose
Mohammed et al. (2010)	8	-	pre	57	180	Single Full dose
Resik et al. (2013)	17	-	pre	96	153	Single Full dose
Resik et al. (2010)	6	-	pre	63	177	Single Full dose
Simasathien et al. (1994)	8	-	pre	40	103	Single Full dose
Zaman et al. (2021)	6	14	post	229	310	Two Full doses
Aziz et al. (2022)	40	48	post	137	137	Two Full doses
Anand et al. (2015)	6	14	pre	142	156	Two Full doses
Snider et al. (2019)	14	22	post	269	271	Two Full doses
Group (2007)	8	16	pre	64	72	Two Full doses
Simoes et al. (1985)	6.5	14.5	pre	62	75	Two Full doses
Simoes et al. (1985)	10	18	pre	20	21	Two Full doses
Simoes et al. (1985)	29	37	pre	18	18	Two Full doses
Bandyopadhyay et al. (2021)	14	36	post	152	154	Two Full doses
Zaman et al. (2021)	14	36	post	302	305	Two Full doses
Zaman et al. (2021)	6	36	post	318	321	Two Full doses
Asturias et al. (2016)	14	36	pre	193	193	Two Full doses
Resik et al. (2020)	16	32	post	25	25	Two Full doses
Resik et al. (2013)	17	34	pre	153	153	Two Full doses
Saleem et al. (2021)	14	-	post	51	125	Single Fractional dose
Ahmad et al. (2022)	6	-	post	27	190	Single Fractional dose
Ahmad et al. (2022)	10	-	post	54	187	Single Fractional dose
Aziz et al. (2022)	40	-	post	107	143	Single Fractional dose
Resik et al. (2020)	16	-	post	13	28	Single Fractional dose
Mohammed et al. (2010)	8	-	pre	31	185	Single Fractional dose
Resik et al. (2010)	6	-	pre	35	187	Single Fractional dose
Resik et al. (2013)	17	-	pre	74	157	Single Fractional dose
Ahmad et al. (2022)	6	14	post	163	190	Two Fractional doses
Aziz et al. (2022)	40	48	post	142	142	Two Fractional doses
Anand et al. (2015)	6	14	pre	123	152	Two Fractional doses
Snider et al. (2019)	6	14	post	173	270	Two Fractional doses
Nirmal et al. (1998)	6	14	pre	21	30	Two Fractional doses
Bandyopadhyay et al. (2021)	14	36	post	190	194	Two Fractional doses
Resik et al. (2020)	16	32	post	26	28	Two Fractional doses
Resik et al. (2013)	17	34	pre	154	157	Two Fractional doses

4.2.1 Full dose model

To the set of N studies, each of which consisted of full doses given at ages d_1^i and d_2^i, to n_i children, of whom s_i, $i = 1, \ldots, N$ were found to be seroconverted (with $d_1 = d_2$ where there was only a single dose) the following model was fit, the linear predictor of which was chosen from a set of models (including those with polynomial predictors) using the Akaike information criterion to select the most parsimonious. The indicator of whether a study had been before or after the 2016 tOPV-bOPV switch was not found to be a useful predictor.

$$s \sim \mathrm{Bin}(n_i, p(d_1^i, d_2^i)), \tag{3}$$

where

$$p(d_1, d_2) = \left(1 + \exp(-(\beta_0 + \beta_1 d_1^i + \beta_2 d_1^{i2} + \beta_3(d_2^i - d_1^i) + \beta_4(d_2^i - d_1^i)^2))\right)^{-1}. \tag{4}$$

The parameter estimates are as follows:

Parameter	estimate	standard deviation
β_0	-2.55	0.161
β_1	0.294	0.0207
β_2	-0.00398	0.000558
β_3	0.294	0.0219
β_4	-0.00384	0.00103.

4.2.2 Fractional dose model

A similar model was used as above, but with

$$p(d_1, d_2) = \left((1 + \exp(-(\gamma_0 + \gamma_1 d_1^i + \gamma_2(d_2^i - d_1^i) + \gamma_3 I(\mathit{Post\ switch}))\right)^{-1}. \tag{5}$$

where $I(\mathit{Post\ switch})$ is an indicator of whether the study was conducted before or after the 2016 Switch.

We then have

Parameter	estimate	standard deviation
γ_0	-1.81	0.123
γ_1	0.0806	0.00636
γ_2	0.292	0.140
γ_3	0.00777	0.110

The fractional dose model uses linear predictors with an additional factor predictor for whether the study had been conducted before or after the Switch from tOPV to bOPV: we did not use the model selected via AIC for fractional doses, as implausible behaviour was displayed at extreme values.

These models enable the prediction of the probability of seroconversion at arbitrary dose ages $d_1 < d_2 < 78$ weeks.

4.3 Immunity estimates 2024-2031

We estimate the population immunity induced by IPV against type-2 poliomyelitis in children under 5 years old at the national level in countries currently administering one or two doses of IPV. For each date in the five years preceding each time point in question, we estimate the probability that a child born on that day is immune. This estimation considers whether the child was eligible and/ or old enough for one or two doses at the date in question, the probability of receiving the doses given the estimated coverage at the scheduled time of administration, and the seroconversion rate at the age of administration.

Schedule and coverage changes during the five years in question are accounted for, with the assumption that RI coverage levels after 2023 remain constant at 2023 levels. For each time point, the constructed probability of being immune is averaged over all children born in the previous five years to estimate the proportion of children with immunity. This assumes an equal age distribution across the cohort.

It is assumed that children are born and age out of the under-five cohort at a constant rate. An "equilibrium position" of immunity is reached five years after any dose introduction or schedule change, having taken effect on all children under five years of age at that point. Uncertainty estimates of immunity are not provided, as the uncertainty on the WUENIC coverage estimate is unknown.

We define $c(t)$, the coverage at time t, d_1, d_2 the scheduled ages at which the first and second doses are to be administered, i_1, i_2 the dates of introductions of doses one and two, $s_1(d)$ the single-dose seroconversion rate for a dose given at age d, $s_2(d, f)$ the two-dose seroconversion rate for doses given at ages d and f. The first dose only coverage, where a two doses are scheduled, is assumed to be $c(d) - c(f)$. We define the following indicator functions for the eligibility and being old enough for particular doses:

$$e_1(t) = I(t + d_1 < i_1), \tag{6}$$

$$e_2(t) = I(t + d_1 < i_2), \tag{7}$$

$$o_1(t, T) = I(T - t > d_1), \tag{8}$$

$$o_2(t, T) = I(T - t > d_2), \tag{9}$$

so that the probability, for $T - 5\text{yrs} < t < T$ that a child born on day t is immune on day T is given by

$$\begin{aligned} p(t, T) = e_2(t)o_2(t, T)\left(s_2(d_1, d_2)c(d_2 + t) + s_1(d_1)(c(d_1 + t) - c(d_2 + t))\right) + \\ (e_1(t)(1 - e_2(t))o_1(t, T)(1 - o_2(t, T)) + \\ e_2(t)o_1(t)(1 - o_2(t)) + \\ e_1(t)(1 - e_2(t))o_2(t, T))s_1(d_1)c(d_1 + t). \end{aligned} \tag{10}$$

Here terms one to four account for

1. Those eligible and old enough for a second dose at time T,
2. Those eligible and old enough for a first dose but not a second dose at time T,
3. Those eligible for a second dose, but only old enough for a first dose at time T,
4. Those old enough for a second dose at time T, but only eligible for the first.

Thus the proportion of children under the age of five with immunity at time T is estimated to be

$$\frac{1}{1826} \sum_{t=(T-1826)}^{T} p(t, T). \tag{11}$$

4.4 Estimates with catch-ups

Where catch-ups were included, the methods used were as above, but with the addition of catch-up coverage K,catch-up date C, and catch-up eligibility indicator

$$e_c(t, T) = o_2(t, T)I(t + d_2 < C). \tag{12}$$

so that the probability, for $T - 5\text{yrs} < t < T$ that a child born on day t is immune on day T is given by

$$p = e_1(t)o_1(t,T)(1-o_2(t,T))(s_1(d_1)c(d_1+t))+$$
$$e_1(t)(1-e_2(t))o_2(t,T)e_c(t,T)((1-K)c(d_1+t)s_1(d_1)+Kc(d_1+t)s_2(d_1,c-t)+K(1-c(d_1+t))s_1(c-t))+$$
$$e_2(t)o_2(t,T)e_c(t,T)((1-K)(c(d_1+t)-c(d_2+t))s_1(d_1)+K(c(d_1+t)-c(d_2+t))s_2(d_1,C-t)+c(d_2+t)s_2(d_1,d_2)+(1-c(d_1+t))Ks_1(C-t))+$$
$$e_2(t)o_2(t,T)(1-e_c(t,T))((c(d_1+t)-c(d_2+t))s_1(d_1)+c(d_2+t)s_2(d_1,d_2)) \quad (13)$$

where terms one two four take into account:

1. Those old enough and eligible for IPV1 (first dose of IPV), but not old enough or eligible for IPV2 (second dose of IPV).
2. Those old enough and eligible for IPV, those not eligible but old enough for IPV2, eligible for catch-up. These are made up of those who have the first dose and no catch-up, the first and catch-up, just catch-up.
3. Those eligible and old enough for first, second dose, and catch-up. These are made up of those who had first dose only, first and catch-up, first and second, just catch-up.
4. those eligible and old enough for both first and second doses, but not catch-up. These are made up of those who had first dose only and those who had both doses.

4.5 Equilibrium immunity with delay.

Distributions of vaccine receipt over time were estimated from Demographic Health Survey (DHS) data. The month of birth of each child is recorded, with the date of the survey and whether a child has received a single dose of DTP-containing vaccine (usually administered at 6 weeks), 3rd dose of DTP vaccine (usually administered at 14 weeks) and the first dose of Measles-containing vaccine (MCV1, usually administered at 39 weeks). Only countries with surveys from 2013 onwards with more than a thousand children surveyed were considered. These are listed below. For each possible delay (in weeks), the immunogenicity at the scheduled age plus the delay was used to construct an immunity estimate. These estimates were then combined into a weighted mean, with weights based on the proportion of children experiencing each specific delay. Delays for first and second IPV doses were assumed independent of one another. The data set derived from DHS survey gives

1. The total number of vaccinated children for each country and vaccine dose (DTP1, DTP3, MCV1).
2. The number of children vaccinated by weeks of age (derived from birth month and vaccination date) as in Derqui et al. (2024).

From these, for each IPV dose, we take the dose (DTP1, DTP3, MCV1) given at the same time or most recently before (this will vary depending on the choice of schedule: current, 6-14, 14-39) and recenter the distribution around the IPV dates, also constructing a joint distribution for which we assume delays on first doses are independent of one another. We remove any (rare) impossible combinations e.g. first doses delayed until after second doses and first doses before birth, and renormalise.

This gives a distribution $p(D_1, D_2) = p(d_1 = D_1, d_2 = D_2)$ for all values $D_1 < D_2 \leq 78$weeks. We use the one and two dose coverage of the scheduled dose ages d_1^*, d_2^*, leading to an immunity estimate of

$$\sum_{D_1=1}^{77} \sum_{D_2=D_1+1}^{78} \left(s_2(D_1,D_2)c(d_2^*) + s_1(D_1)(c(d_1^*) - c(d_2^*))\right) p(D_1,D_2) \quad (14)$$

Delay data were available for the following countries:

- **African Region:** Angola, Benin, Burundi, Chad, Democratic Republic of the Congo, Gabon, Gambia, Ghana, Guinea, Kenya, Lesotho, Liberia, Madagascar, Malawi, Mali, Mauritania, Namibia, Nigeria, Rwanda, Senegal, Sierra Leone, Togo, Uganda, United Republic of Tanzania, Zimbabwe
- **Americas Region:** Guatemala, Haiti
- **South-East Asia Region:** Bangladesh, Indonesia, Maldives, Myanmar, Timor-Leste
- **Eastern Mediterranean Region:** Pakistan, Yemen
- **Western Pacific Region:** Philippines
- **European Region:** Tajikistan

5 Additional Figures

5.1 Delay in vaccine administration

Figure A shows the distribution of age of receipt of DTP1 and MCV1 in Angola, Nigeria and Pakistan, according to data from the Demographic and health survey, and the estimated equilibrium positions of immunity for countries for which delay data was available, on a six-fourteen or fourteen-thirty nine week schedule, for comparison of schedules both when delay is and is not accounted for. It can be seen that when there is no delay, the 14/39 week schedule is almost universally better, while when delay is accounted for, this difference is lessened somewhat.

5.2 Coverage decline (WUENIC 2023) in countries considered

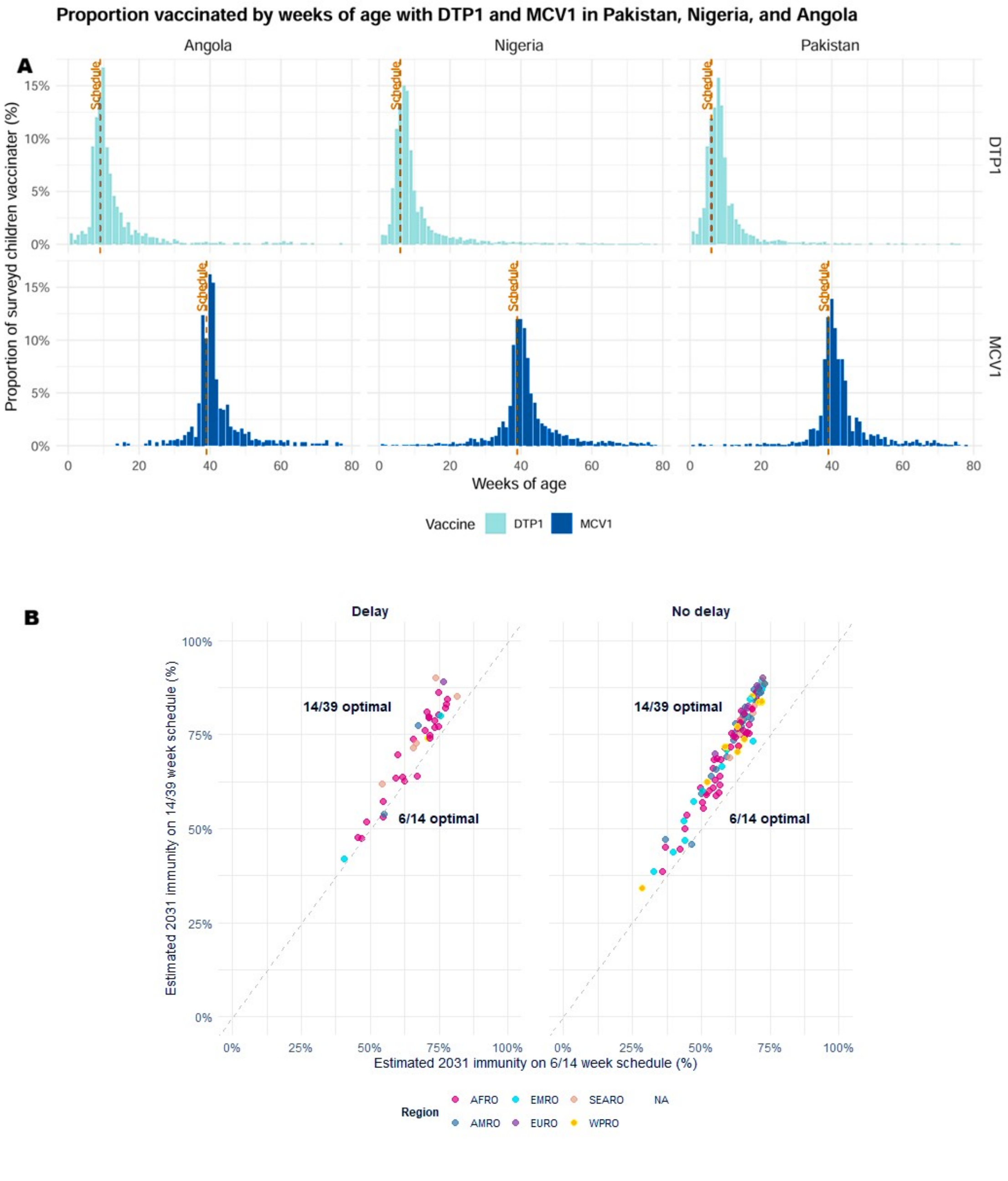

Figure A: Projected type-two population immunity induced by two doses of IPV with and without accounting for delay in routine immunisation. A) Estimated vaccination delay distributions of for first dose of Diphtheria, Tetanus, and Pertussis vaccine (DTP1) in Nigeria and Pakistan and first dose of measles vaccine (MCV1) from Demographic and Health surveys, B) Comparison of schedules when vaccination delay is and is not accounted for.

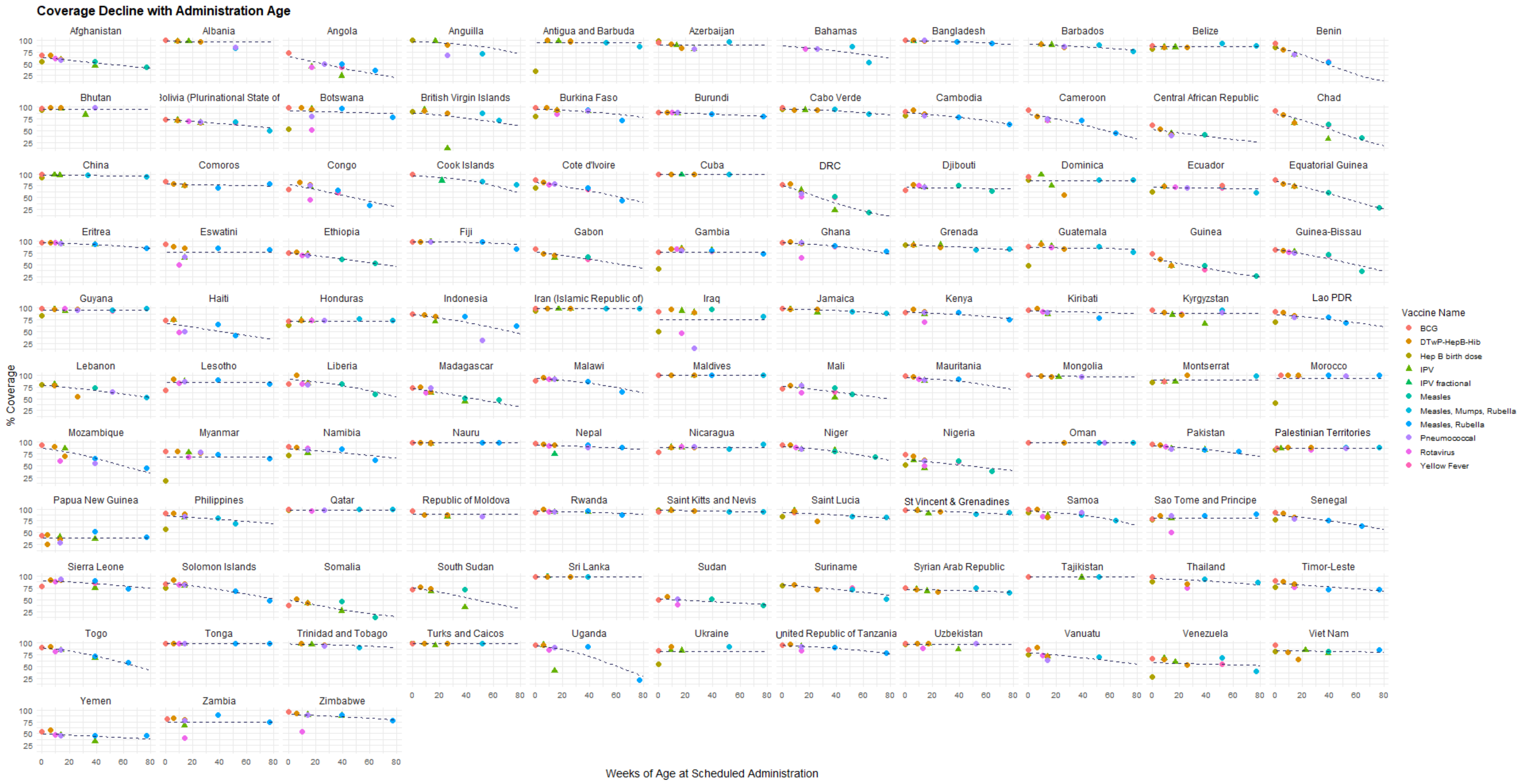

Figure B: 2023 Estimates of coverage, plotted against age of scheduled administration. WUENIC estimates used where available, administrative coverage is used for Anguilla, Turks and Caicos, Montserrat, and the British Virgin Islands.

6 Number of countries administering different IPV dose numbers per year

Table Y: The number of the 134 bOPV-using countries and territories administering each number of IPV doses in routine immunisation for the full year (as of 2025): the dose must have been introduced at least by 31st December the previous year to be counted.

	2017	2018	2019	2020	2021	2022	2023	2024	2025
No doses	22	17	1	0	0	0	0	0	0
1 dose	89	89	101	95	90	69	52	40	27
2 doses	21	20	20	27	32	50	64	70	82
3 doses	0	0	4	4	4	7	10	10	11
4 doses	0	0	0	0	0	0	0	5	5
5 doses	0	2	2	2	2	2	2	3	3
2 fractional	2	6	6	6	6	6	6	5	5
3 fractional	0	0	0	0	0	0	0	1	1

References

Mohammad Ahmad, Harish Verma, Jagadish Deshpande, Abhishek Kunwar, Ashish Bavdekar, Niranjana S Mahantashetti, Balasundaram Krishnamurthy, Manish Jain, Mannancheril A Mathew, Shailesh D Pawar, et al. Immunogenicity of fractional dose inactivated poliovirus vaccine in india. *Journal of the Pediatric Infectious Diseases Society*, 11(2):60–68, 2022.

Abhijeet Anand, K Zaman, Concepción F Estívariz, Mohammad Yunus, Howard E Gary, William C Weldon, Tajul I Bari, M Steven Oberste, Steven G Wassilak, Stephen P Luby, et al. Early priming with inactivated poliovirus vaccine (ipv) and intradermal fractional dose ipv administered by a microneedle device: A randomized controlled trial. *Vaccine*, 33(48):6816–6822, 2015.

Edwin J Asturias, Ananda S Bandyopadhyay, Steve Self, Luis Rivera, Xavier Saez-Llorens, Eduardo Lopez, Mario Melgar, James T Gaensbauer, William C Weldon, M Steven Oberste, et al. Humoral and intestinal immunity induced by new schedules of bivalent oral poliovirus vaccine and one or two doses of inactivated poliovirus vaccine in latin american infants: an open-label randomised controlled trial. *The Lancet*, 388 (10040):158–169, 2016.

Asma B Aziz, Harish Verma, Visalakshi Jeyaseelan, Mohammad Yunus, Samarea Nowrin, Deborah D Moore, Bernardo A Mainou, Ondrej Mach, Roland W Sutter, and Khalequ Zaman. One full or two fractional doses of inactivated poliovirus vaccine for catch-up vaccination in older infants: A randomized clinical trial in bangladesh. *The Journal of Infectious Diseases*, 226(8):1319–1326, 2022.

Ananda S Bandyopadhyay, Chris Gast, Luis Rivera, Xavier Sáez-Llorens, M Steven Oberste, William C Weldon, John Modlin, Ralf Clemens, Sue Ann Costa Clemens, Jose Jimeno, et al. Safety and immunogenicity of inactivated poliovirus vaccine schedules for the post-eradication era: a randomised open-label, multicentre, phase 3, non-inferiority trial. *The Lancet Infectious Diseases*, 21(4):559–568, 2021.

Nieves Derqui, Isobel M Blake, Elizabeth J Gray, Laura V Cooper, Nicholas C Grassly, Margarita Pons-Salort, and Katy A M Gaythorpe. Timeliness of 24 childhood immunisations and evolution of vaccination delay: Analysis of data from 54 low- and middle-income countries. *PLOS Glob Public Health*, 4(11):e0003749, 2024.

Nicholas C Grassly. Immunogenicity and effectiveness of routine immunization with 1 or 2 doses of inactivated Poliovirus vaccine: systematic review and meta-analysis. *The Journal of infectious diseases*, 210(suppl_1):S439–S446, 2014.

Cuba IPV Study Collaborative Group. Randomized, placebo-controlled trial of inactivated poliovirus vaccine in cuba. *New England Journal of Medicine*, 356(15):1536–1544, 2007.

N Linder, M Yaron, R Handsher, J Kuint, E Birenbaum, R Mazkereth, D Lubin, E Mendelson, O Safrir, and B Reichman. Early immunization with inactivated poliovirus vaccine in premature infants. *The Journal of pediatrics*, 127(1):128–130, 1995.

A Marshall McBean, Mary Lou Thoms, Paul Albrecht, Judith C Cuthie, Roger Bernier, Field Staff, and Coordinating Committee. Serologic response to oral polio vaccine and enhanced-potency inactivated polio vaccines. *American journal of epidemiology*, 128(3):615–628, 1988.

Ali Jafer Mohammed, Salah AlAwaidy, Shyam Bawikar, Padmamohan J Kurup, Emadaldin Elamir, Mahmoud MA Shaban, Sharif M Sharif, Harrie GAM van der Avoort, Mark A Pallansch, Pradeep Malankar, et al. Fractional doses of inactivated poliovirus vaccine in oman. *New England Journal of Medicine*, 362(25):2351–2359, 2010.

Stephen Nirmal, Thomas Cherian, Benjamin U Samuel, J Rajasingh, P Raghupathy, and T Jacob John. Immune response of infants to fractional doses of intradermally administered inactivated poliovirus vaccine. *Vaccine*, 16(9-10):928–931, 1998.

Sonia Resik, Alina Tejeda, Pedro Mas Lago, Manuel Diaz, Ania Carmenates, Luis Sarmiento, Nilda Alemani, Belkis Galindo, Anthony Burton, Martin Friede, et al. Randomized controlled clinical trial of fractional doses of inactivated poliovirus vaccine administered intradermally by needle-free device in cuba. *The Journal of infectious diseases*, 201(9):1344–1352, 2010.

Sonia Resik, Alina Tejeda, Roland W Sutter, Manuel Diaz, Luis Sarmiento, Nilda Alemañi, Gloria Garcia, Magilé Fonseca, Lai Heng Hung, Anna-Lea Kahn, et al. Priming after a fractional dose of inactivated poliovirus vaccine. *New England Journal of Medicine*, 368(5):416–424, 2013.

Sonia Resik, Ondrej Mach, Alina Tejeda, Visalakshi Jeyaseelan, Magile Fonseca, Manuel Diaz, Nilda Alemany, Lai Heng Hung, Yoan Aleman, Ileana Mesa, et al. Immunogenicity of intramuscular fractional dose of inactivated poliovirus vaccine. *The Journal of Infectious Diseases*, 221(6):895–901, 2020.

Ali Faisal Saleem, Ondrej Mach, Mohammad Tahir Yousafzai, Zaubina Kazi, Attaullah Baig, Muhammad Sajid, Vishali Jeyaseelan, Roland W Sutter, and Anita KM Zaidi. One-year decline of poliovirus antibodies following fractional-dose inactivated poliovirus vaccine. *The Journal of infectious diseases*, 223(7):1214–1221, 2021.

Sriluck Simasathien, Sricharoen Migasena, Coen Beuvery, Gijsbert Van Steenis, Rudiwilai Samakoses, Punnee Pitisuttitham, and Timo Vesikari. Comparison of enhanced potency inactivated poliovirus vaccine (eipv) versus standard oral poliovirus vaccine (opv) in thai infants. *Scandinavian journal of infectious diseases*, 26(6):731–738, 1994.

Eric AF Simoes, Baby Padmini, Mark C Steinhoff, Malati Jadhav, and T Jacob John. Antibody response of infants to two doses of inactivated poliovirus vaccine of enhanced potency. *American journal of diseases of children*, 139(10):977–980, 1985.

Cynthia J Snider, Khalequ Zaman, Concepcion F Estivariz, Mohammad Yunus, William C Weldon, Kathleen A Wannemuehler, M Steven Oberste, Mark A Pallansch, Steven GF Wassilak, Tajul Islam A Bari, et al. Immunogenicity of full and fractional dose of inactivated poliovirus vaccine for use in routine immunisation and outbreak response: an open-label, randomised controlled trial. *The Lancet*, 393 (10191):2624–2634, 2019.

Beckie N Tagbo, Harish Verma, Zubairu M Mahmud, Kolade Ernest, Roosevelt O Nnani, Chinedu Chukwubike, Kehinde T Craig, Abdullahi Hamisu, William C Weldon, Steven M Oberste, et al. Randomized controlled clinical trial of bivalent oral poliovirus vaccine and inactivated poliovirus vaccine in nigerian children. *The Journal of infectious diseases*, 226(2):299–307, 2022.

World Health Organisation. Immunisation data portal. `https://immunizationdata.who.int/`. Accessed 16-10-2023.

Khalequ Zaman, Stephanie D Kovacs, Kristin Vanderende, Asma Aziz, Mohammed Yunus, Sara Khan, Cynthia J Snider, Qian An, Concepcion F Estivariz, M Steven Oberste, et al. Assessing the immunogenicity of three different inactivated polio vaccine schedules for use after oral polio vaccine cessation, an open label, phase iv, randomized controlled trial. *Vaccine*, 39(40):5814–5821, 2021.
