# Supplementary material for: Estimating population immunity against serotype-two poliomyelitis from the inactivated polio vaccine in routine immunization across 112 countries: A modelling study
Source: PLoS Med. 2026 Mar 19;23(3):e1004952. doi: 10.1371/journal.pmed.1004952 (PMC13001910; doi:10.1371/journal.pmed.1004952)
Supplement: Attachment — Submitted filename: Plos.docx [file pmed.1004952.s003.docx]

This is a well structured and written manuscript. These are the specific strengths of the manuscript

Highly topical; directly addresses challenges in polio eradication and IPV implementation post-OPV2 withdrawal.

Solid combination of empirical data and cohort modeling; integrates real-world coverage and delay metrics.

Covers 114 countries with stratified modeling scenarios.

However, these are the minor suggestions that could be considered:

The study would be strengthened with confidence intervals or probabilistic sensitivity analyses around projected immunity estimates.

Ignoring serotype-2 immunity from outbreak response campaigns underestimates total population immunity and risks.

The text is dense in some sections. Figures are highly detailed but may overwhelm readers; consider supplemental simplification or policy briefs.
