# Supplementary material for: Estimating population immunity against serotype-two poliomyelitis from the inactivated polio vaccine in routine immunization across 112 countries: A modelling study
Source: PLoS Med. 2026 Mar 19;23(3):e1004952. doi: 10.1371/journal.pmed.1004952 (PMC13001910; doi:10.1371/journal.pmed.1004952)
Supplement: Attachment — Submitted filename: IPV_response_to_reviewers.docx [file pmed.1004952.s005.docx]

*Reviewer #1 (Statistics):
This is an obviously important topic, and the logic of the study is robust and the reporting is detailed. While this is based on observational data, it is possible to be the most reliable data, given that relevant trial data is not and probably will not be available.

However, I have several comments, some of which might be important to the robustness of the findings:

1. In the models that estimate the coverage decline and seroconversion by age, it seems that the primary predictors (e.g. week/age) were all modelled assuming linear relationship. While the model fit with data points on main manuscript p6 appear ok, it'd be good to check for nonlinearity by e.g. fitting fractional polynomials or splines to ensure these won't affect the conclusions.*

Thank you, while in general we agree that such an approach would be optimal, the number of available data points (WUENIC estimates for the number of RI vaccines) for each country (N = 4 – 11) does not justify the additional complexity without severe overfitting. We have now included plots in the Supplementary Materials of routine immunisation coverage by age for every country and the reviewer will see that our assumption of a linear relationship is reasonable for the number of available data points.

Additionally, we have included a plot of coverage decline with age of administration (an expanded version of Figure 3) for all countries considered in the Supplementary Materials:

 *2. Given the observational nature and the various assumptions behind the models, I wondered if the interpretation is too strong. I'd suggest to at least adding 'under current estimation' before the interpretations in the Abstract.*

Thank you for this suggestion, we have altered the abstract to contain the above, as follows:

**Interpretation:** Under these estimates, IPV schedules and coverage are suboptimal in many countries. Those with a single dose should introduce a second on the 14-39 week schedule; those on early schedules would benefit from adopting the 14-39 week schedule. IPV catch-up campaigns are recommended where RI coverage is low

*3. Similarly, won't it be a limitation that the estimates were based on observational data and confounding is possible.*

Our estimates of population immunity are informed by both i) a review of immunogenicity data by age from multiple clinical trials; and ii) national routine immunisation coverage as a function of age (WUENIC). The latter is informed by multiple data sources and expert opinion. It is more reliable than administrative data but is not a perfect data source. We have acknowledged the limitations of national coverage estimates in the discussion:

Future research should address this in collaboration with local healthcare providers. When considering coverage, WUENIC are informed by multiple data sources and expert opinion. They are more reliable than administrative data but are not a perfect data source, nor, being whole-country estimates, can they capture subnational heterogeneity, which may be an important driver of polio risk where pockets of very low coverage exist [28] It is a further limitation that we do not address this subnational heterogeneity, which, for example has been demonstrated in South Africa [29] and is likely substantial in many countries. In such places smaller scale targeted interventions may be more appropriate or cost-effective. This is particularly pertinent where areas have greater Polio exposure.

*Reviewer #2:
This is a very interesting paper that used a modelling approach to estimate poliovirus type 2 (PV2) immunity in under-5 children in 114 countries using bOPV in routine immunization. They conducted a systematic review of studies on PV2 seroconversion by age of IPV administration (with one or two doses and with full and fractional doses); then used a binomial regression model to fit seroconversion as a function of age at the time of the first dose and the time interval between doses; finally, considering also countries' data on routine immunization coverage as a function of age and data on doses delays, they estimated  immunity of under-5 children to PV2 in each country, in 2025 and 2030. The results demonstrate median PV2 immunity of only 60% in 2025, that would increase to only 66% in 2030, if the vaccination schedule of the country is kept the same (with 19 countries with PV2 immunity <50% in 2030). The results point out that, besides the number of IPV doses and vaccine
coverage, the age of IPV administration is important to achieve better PV2 immunity. The paper results may help public health managers to choose the best schedule to achieve high population immunity to PV2.

The study was well conducted, and the paper is well written. I did not have any suggestions to improve it.*

Thank you very much for your review.  *Reviewer #3:
The approach taken is attractive in its data driven nature; although there are many data challenges, the authors have taken the time to organise what data there are to maximise its usefulness - and the combination of a simple/robust but still mathematically useful approach to estimating immunity levels.*

Thank you very much for your review. *Reviewer #4:
The model really is very simple. It is little more than multiplying the country-specific coverage by the efficacy. This does mean that there is little to go wrong, though. My only real criticisms are these:

1) There is no attempt to check (or validate) the model. Many serological surveys have been undertaken over the years, and some comparison of these to the estimated seroprevalence, should really be done. A much weaker, but also interesting check would be to compare the risk of a cVDPV outbreak with the estimated coverage.*

Thank you very much for your review. This is indeed a limitation, however, while we considered validation with serosurveys we found three major barriers to this being a useful approach:

- Firstly, many serosurveys are carried out either too soon after the end of routine use of tOPV to be relevant, e.g. (2), or following an outbreak response campaign with type 2 OPV, e.g. (4),(6), meaning they are not a useful measure of routine-only IPV use. One study (7) conducted after a WPV1 outbreak in Papua New Guinea had not had recent type 2 OPV use, however this was only conducted over a small number of easily accessible provinces, the estimated type two seroprevalence rate of which was higher than even the WUENIC whole-country coverage estimate for 2023 and is not reflective of the coverage levels of the whole country.
- Secondly, multiple seroprevalence studies [(1), (2), (3) (7)] were conducted prior to the time period that we predict IPV population immunity from and the rapidly changing schedules and recent second dose introductions mean that we cannot compare the results of the seroprevalence surveys to our predictions.
- Finally, the surveys tend to require presentation at a clinic or health centre, as in (2),(3), which makes the sample unrepresentative of the whole country in locations where coverage and health system engagement are low, e.g. (2) recruited participants who presented at a health centre for treatment of minor illnesses OR to receive immunisation.

Similarly, IPV-induced immunity is a poor predictor of the risk of a cVDPV outbreak, primarily as it does not inhibit transmission, instead protecting against symptoms. Secondly, it would be virtually impossible in nearly every relevant context to disentangle effect from IPV-induced immunity from effect of OPV-induced immunity, as used in outbreak response.

As validation via serosurvey may become an option in future (dependent on how future serosurveys are carried out, we have included the following in the discussion;

“Future work could consider validation of the approach used in this study using the results of seroprevalence surveys e.g. [30-32]. This has not yet been feasible as many prior surveys are limited in relevance due to their timing around tOPV cessation or OPV2 outbreak responses, being carried out in the context of now obsolete IPV schedules, or may include sampling bias from requiring at-clinic presentation in otherwise low coverage settings.”

***References***

1. *de Deus N, Capitine IPU, Bauhofer AFL, Marques S, Cassocera M, Chissaque A, et al. Immunogenicity of Reduced-Dose Monovalent Type 2 Oral Poliovirus Vaccine in Mocuba, Mozambique. J Infect Dis. 2022;226(2):292–8. doi:10.1093/infdis/jiaa704*
2. *Hussain, I., Shah, M., Ahmed, T., et al. (2018). Seroprevalence of anti-polio antibodies in children from polio high risk area of Afghanistan: A cross sectional survey 2017. Vaccine, 36(15), 1921–1924.* [*https://doi.org/10.1016/j.vaccine.2018.02.096*](https://doi.org/10.1016/j.vaccine.2018.02.096)
3. *Hussain, I., Alam, M. M., Ayub, M., et al. (2024). Exploring the path to polio eradication: Insights from consecutive seroprevalence surveys among Pakistani children. Frontiers in Public Health, 12, Article 1384410.* [*https://doi.org/10.3389/fpubh.2024.1384410*](https://doi.org/10.3389/fpubh.2024.1384410)
4. *Kennedy, S. B., Scharff, D. P., Kollie, K., et al. (2023). Poliovirus antibodies following two rounds of campaigns with a type 2 novel oral poliovirus vaccine in Liberia: A clustered, population-based seroprevalence survey. The Lancet Global Health, 11(6), e917–e923.* [*https://doi.org/10.1016/S2214-109X(23)00122-7*](https://doi.org/10.1016/S2214-109X(23)00122-7)
5. *Mirzoev, A., Raval, M., Henao-Restrepo, A. M., et al. (2022). Assessment of serological responses following vaccination campaigns with type 2 novel oral polio vaccine: A population-based study in Tajikistan in 2021. The Lancet Global Health, 10(12), e1807–e1814.* [*https://doi.org/10.1016/S2214-109X(22)00325-2*](https://doi.org/10.1016/S2214-109X(22)00325-2)
6. *Sharma, A. K., Bahl, S., Estívariz, C. F., et al. (2023). Persistence of immunity following a single dose of inactivated poliovirus vaccine: A phase 4, open-label, non-randomised clinical trial. The Lancet Microbe, 4(11), e923–e930.* [*https://doi.org/10.1016/S2666-5247(23)00123-4*](https://doi.org/10.1016/S2666-5247(23)00123-4)
7. Pomat, W., Lopez Cavestany, R., Jeyaseelan, V., Ford, R., Gare, J., Avagyan, T., Grabovac, V., Bettels, D., Mekonnen, D., Jones, K. A. V., Mainou, B. A., & Mach, O. (2023). Poliovirus serological assay after the cVDPV1 outbreak in Papua New Guinea: A cross-sectional study from 2020 to 2021. The Lancet Regional Health – Western Pacific, 44, 100986. <https://doi.org/10.1016/j.lanwpc.2023.100986>

*2) bOPV use is not taken into account. Hundreds of millions (even billions?) of doses of mOPV2 and nOPV2 have been used since 2016. The effect of IPV campaigns is taken into account. I don't understand why OPV campaigns are not also taken into account.*

We did not consider bOPV as it does not offer any immunity from type-two poliomyelitis.

We have not included type-two OPV-induced immunity from outbreak response for several reasons. Firstly, IPV induced immunity from routine immunisation is an important measure of how robust the routine, permanent health systems are to outbreaks of poliovirus, separate from emergency responses which produce only a temporary increase in immunity. Such a measure is particularly important for countries which may be considering near-future switch to an IPV-only schedule ( as have e.g. Turkmenistan, Paraguay in recent years). For type two poliovirus specifically, IPV represents a "safety net" in the context of continuing eradication efforts. On a more practical note, no reliable data on coverage is collected for outbreak response, making the inclusion of OPV-induced immunity from campaigns unfeasible. We have included the following in the introduction to make this clearer for the reader:

“Despite some increased immunity in selected locations due to outbreak response activities, which is temporary as children age out of the under-five cohort, immunity from inactivated polio vaccine in routine immunisation now represents the only long-term routinely-provided protection against type-two poliomyelitis.”

*3) Clarke and Sanderson (Lancet 2009) should be referenced, when taking into account delays to vaccination. The method you use is based on this. There are also other papers that predate the reference that you do give (e.g. doi: 10.1136/jech.2010.124651.)*

Thank you, we were not previously aware of this. We have now included the citation.

[15] Andrew Clark and Colin Sanderson. Timing of children’s vaccinations in 45 low-income and middle-income countries: an analysis of survey data. The Lancet, 373(9674):1543–1549, 2009

*Reviewer #5:
The study would be strengthened with confidence intervals or probabilistic sensitivity analyses around projected immunity estimates.*

Thank you for your review. We agree that adding these would have been beneficial, however we are limited by the fact that various sources of the data (e.g. WUENIC routine immunisation coverage) are themselves estimates provided without uncertainty, meaning it would be impossible to provide meaningful uncertainty estimates to our own – any would likely be an underestimate of uncertainty and give misleading and unsubstantiated confidence to our results.

We have clarified this as a limitation in the discussion:

“We have not given uncertainty estimates for population immunity because many of the data points lack uncertainty estimates (e.g. WUENIC routine immunisation coverage estimates), and thus any derived uncertainty estimates which assume these to be known constants may be misleading.”

*Ignoring serotype-2 immunity from outbreak response campaigns underestimates total population immunity and risks.*

Thank you for your comment. We have not included OPV-induced immunity from outbreak response for several reasons. Firstly, IPV induced immunity from routine immunisation is an important measure of how robust the routine, permanent health systems are to outbreaks of poliovirus, separate from emergency responses which produce only a short term increase in immunity in the under 5 year population as a whole. Such a measure is particularly important for certain countries which may be considering near-future switch to an IPV-only schedule (as have e.g. Turkmenistan, Paraguay in recent years). For type two poliovirus specifically, IPV represents a "safety net" in the context of continuing eradication efforts. On a more practical note, no reliable data on coverage is systematically collected for outbreak response, making the inclusion of OPV-induced immunity from campaigns unfeasible. We have included the following in the introduction to clarify for the reader:

“Despite some increased immunity in selected locations due to outbreak response activities, which is temporary as children age out of the under-five cohort, immunity from inactivated polio vaccine in routine immunisation now represents the only long-term routinely-provided protection against type-two poliomyelitis.”

*The text is dense in some sections. Figures are highly detailed but may overwhelm readers; consider supplemental simplification or policy briefs.*

Thank you for this very helpful suggestion. We have now written this policy ‘box’ which we propose to the editor can be included in the manuscript.:

**Author summary**

• Multiple vaccine-derived type-2 poliovirus outbreaks continue to circulate.

• The inactivated polio vaccine (IPV) is the only vaccine delivered through routine immunization that protects against type-2 poliomyelitis. A minimum of two IPV doses is recommended to be given at 14 weeks of age and 4 months later, (e.g. 39 weeks), although 43 countries or territories administer an earlier week schedule, often at 6/14 weeks.

• There is a need to quantify type-2 population immunity from IPV given differences in country schedules, national coverage and the timing of the second dose introduction.

**What was done:**

• We developed a model to estimate national population immunity against type-2 poliomyelitis across 112 countries in children < 5 years old.

• This approach combines country specific declines in routine vaccination coverage by age, with a review of increased seroconversion by age of IPV (inactivated polio vaccine) administration.

• We then quantified the value of catch-up vaccination campaigns to increase immunity levels.

**Key Findings and policy recommendations:**

• The 14 / 39 week schedule of IPV administration is preferable to the earlier 6/14 week alternative schedule across all countries meaning up to 40 countries or territories would benefit in transitioning to the later schedule to avoid leaving a substantial proportion of children under the age of five years vulnerable to poliomyelitis.

• The 23 countries still using only one dose of IPV in routine immunisation should urgently introduce a second dose on the 14/39-week schedule.

• Current IPV-induced immunity levels are critically low in many locations (25 countries or territories < 50% population immunity in children under five years of age).

• Second dose introduction and schedule optimisation may not be enough to achieve high immunity in certain locations: 8 countries overall would still have immunity < 50% by 2031 due to low routine immunisation coverage.

• Catch-up campaigns could be considered to shorten the time taken to reach higher immunity in countries with recent introductions of a second dose of IPV, and in countries where routine IPV coverage is very low and immunity would otherwise remain critically low.

*General editorial requests:
(Note: not all will apply to your paper, but please check each item carefully and include your reponses as part of your point-by-point, noting N/A where appropriate)

*       We ask every co-author listed on the manuscript to fill in a contributing author statement, making sure to declare all competing interests. If any of the co-authors have not filled in the statement, we will remind them to do so when the paper is revised. If all statements are not completed in a timely fashion this could hold up the re-review process. If new competing interests are declared later in the revision process, this may also hold up the submission. Should there be a problem getting one of your co-authors to fill in a statement we will be in contact. Please do not add or remove authors without first discussing this with the handling editor. You can see our competing interests policy here:* [*http://journals.plos.org/plosmedicine/s/competing-interests*](http://journals.plos.org/plosmedicine/s/competing-interests)*.

*       Please upload any figures associated with your paper as individual TIF or EPS files with 300dpi resolution at resubmission; please read our figure guidelines for more information on our requirements:* [*http://journals.plos.org/plosmedicine/s/figures*](http://journals.plos.org/plosmedicine/s/figures)*. While revising your submission, please upload your figure files to the PACE digital diagnostic tool,* [*https://pacev2.apexcovantage.com/*](https://pacev2.apexcovantage.com/)*. PACE helps ensure that figures meet PLOS requirements. To use PACE, you must first register as a user. Then, login and navigate to the UPLOAD tab, where you will find detailed instructions on how to use the tool. If you encounter any issues or have any questions when using PACE, please email us at* [*PLOSMedicine@plos.org*](mailto:PLOSMedicine@plos.org)*.*

*DONE*

**       Please ensure that the paper adheres to the PLOS Data Availability Policy (see* [*http://journals.plos.org/plosmedicine/s/data-availability)*](http://journals.plos.org/plosmedicine/s/data-availability))*, which requires that all data underlying the study's findings be provided in a repository or as Supporting Information. For data residing with a third party, authors are required to provide instructions with contact information (web or email address) for obtaining the data. Please note that a study author cannot be the contact person for the data. PLOS journals do not allow statements supported by "data not shown" or "unpublished results." For such statements, authors must provide supporting data or cite public sources that include it.*

*This will be provided in a repository*

**       We expect all researchers with submissions to PLOS in which author-generated code underpins the findings in the manuscript to make all author-generated code available without restrictions upon publication of the work. In cases where code is central to the manuscript, we may require the code to be made available as a condition of publication. Authors are responsible for ensuring that the code is reusable and well documented. Please make any custom code available, either as part of your data deposition or as a supplementary file. Please add a sentence to your data availability statement regarding any code used in the study, e.g. "The code used in the analysis is available from Github [URL] and archived in Zenodo [DOI link]" Please review our guidelines at* [*https://journals.plos.org/plosmedicine/s/materials-software-and-code-sharing*](https://journals.plos.org/plosmedicine/s/materials-software-and-code-sharing) *and ensure that your code is shared in a way that follows best practice and facilitates reproducibility and reuse. Because Github depositions can*
*be readily changed or deleted, we encourage you to make a permanent DOI'd copy (e.g. in Zenodo) and provide the URL.*

*This will be provided in a repository*

**       Please ensure that the study is reported according to the STROBE guideline (or appropriate extension) and include the completed STROBE checklist as Supporting Information. When completing the checklist, please use section and paragraph numbers, rather than page numbers. Please add the following statement, or similar, to the Methods: "This study is reported as per STROBE guideline (S1 Checklist).”*

*NA This is not a cross sectional or cohort study*

*FORMATTING - GENERAL*
**       Abstract: Please structure your abstract using the PLOS Medicine headings (Background, Methods and Findings, Conclusions). Please combine the Methods and Findings sections into one section.*

*DONE*

**       At this stage, we ask that you include a short, non-technical Author Summary of your research to make findings accessible to a wide audience that includes both scientists and non-scientists. The Author Summary should immediately follow the Abstract in your revised manuscript. This text is subject to editorial change and should be distinct from the scientific abstract. Ideally each sub-heading should contain 2-3 single sentence, concise bullet points containing the most salient points from your study. In the final bullet point of 'What Do These Findings Mean?', please include the main limitations of the study in non-technical language. Please see our author guidelines for more information:* [*https://journals.plos.org/plosmedicine/s/revising-your-m**anuscript#loc-author-summary*](https://journals.plos.org/plosmedicine/s/revising-your-manuscript#loc-author-summary)*.*
DONE

**       Please express the main results with 95% CIs as well as p values. When reporting p values please report as p<0.001 and where higher as the exact p value p=0.002, for example. Throughout, suggest reporting statistical information as follows to improve clarity for the reader "22% (95% CI [13%,28%]; p</=)". Please be sure to define all numerical values at first use.*
*DONE WHERE POSSIBLE / APPLICABLE – where not possible, explanation has been provided.*

**       Please include page numbers and line numbers in the manuscript file. Use continuous line numbers (do not restart the numbering on each page).*

DONE

**       Please cite the reference numbers in square brackets. Citations should precede punctuation.*

*Done*

*FIGURES AND TABLES DONE*
**       Please provide titles and legends for all figures and tables (including those in Supporting Information files).*

**       Please define all abbreviations used in each figure/table (including those in Supporting Information files).*

**       Please consider avoiding the use of red and green in order to make your figure more accessible to those with colour blindness.*

*SUPPLEMENTARY MATERIAL*
**       Please note that supplementary material will be posted as supplied by the authors. Therefore, please amend it according to the relevant comments outlined here.*

*DONE*

**       Please cite your Supporting Information as outlined here:* [*https://journals.plos.org/plosmedicine/s/supporting-information*
DONE](https://journals.plos.org/plosmedicine/s/supporting-informationDONE)
*REFERENCES*
**       PLOS uses the numbered citation (citation-sequence) method and first six authors, et al.*

*DONE*

**       Please ensure that journal name abbreviations match those found in the National Center for Biotechnology Information (NCBI) databases (*[*http://www.ncbi.nlm.nih.gov/nlmcatalog/journals*](http://www.ncbi.nlm.nih.gov/nlmcatalog/journals)*), and are appropriately formatted and capitalised.*

*DONE*

**       Where website addresses are cited, please include the complete URL and specify the date of access (e.g. [accessed: 12/06/2023]).*

*DONE*

**       Please also see* [*https://journals.plos.org/plosmedicine/s/submission-guidelines#loc-references*](https://journals.plos.org/plosmedicine/s/submission-guidelines#loc-references) *for further details on reference formatting.*

*DONE*

*OBSERVATIONAL STUDIES – Not specifically relevant*

**       Abstract: Please include the study design, population and setting, number of participants, years during which the study took place (enrollment and follow up), length of follow up, and main outcome measures.*

**       Please ensure that the study is reported according to the STROBE (or appropriate STOBE extension; eg, RECORD) guideline (available from:* [*https://www.equator-network.org/reporting-guidelines/strobe)*](https://www.equator-network.org/reporting-guidelines/strobe)) *and include the completed STROBE (or STROBE extension) checklist as Supporting Information. Please add the following statement, or similar, to the Methods: "This study is reported as per the Strengthening the Reporting of Observational Studies in Epidemiology (STROBE) guideline (S1 Checklist)." When completing the checklist, please use section and paragraph numbers, rather than page numbers.*
**       For all observational studies, in the manuscript text, please indicate: (1) the specific hypotheses you intended to test, (2) the analytical methods by which you planned to test them, (3) the analyses you actually performed, and (4) when reported analyses differ from those that were planned, transparent explanations for differences that affect the reliability of the study's results. If a reported analysis was performed based on an interesting but unanticipated pattern in the data, please be clear that the analysis was data driven.*

**       Please state in the Methods section whether the study had a prospective protocol or analysis plan. If a prospective analysis plan (from your funding proposal, IRB or other ethics committee submission, study protocol, or other planning document written before analyzing the data) was used in designing the study, please include the relevant document(s) with your revised manuscript as a Supporting Information file to be published alongside your study and cite it in the Methods section. A legend for this file should be included at the end of your manuscript. If no such document exists, please make sure that the Methods section transparently describes when analyses were planned, and when/why any data-driven changes to analyses took place. Changes in the analysis, including those made in response to peer review comments, should be identified as such in the Methods section of the paper, with rationale.*

*MODELLING STUDIES*
NA our manuscript does not implement a mathematical transmission model
*The following list is derived from Geoffrey P Garnett, Simon Cousens, Timothy B Hallett, Richard Steketee, Neff Walker. Mathematical models in the evaluation of health programmes. (2011) Lancet DOI:10.1016/S0140-6736(10)61505-X:*   
               
**       If pertinent, please provide a diagram that shows the model structure, including how the natural history of the disease is represented, the process and determinants of disease acquisition, and how the putative intervention could affect the system.*

*Not relevant for our approach.*

**       Please provide a complete list of model parameters, including clear and precise descriptions of the meaning of each parameter, together with the values or ranges for each, with justification or the primary source cited and important caveats about the use of these values noted.*

This has been done where appropriate (only in the supplementary information)

**       Please provide a clear statement about how the model was fitted to the data, including goodness-of-fit measure, the numerical algorithm used, which parameter varied, constraints imposed on parameter values, and starting conditions.*

*Not relevant for our particular approach.*

**       For uncertainty analyses, please state the sources of uncertainties quantified and not quantified [can include parameter, data, and model structure].*

*This has been done where appropriate.*

**       Please provide sensitivity analyses to identify which parameter values are most important in the model. Uncertainty estimates seek to derive a range of credible results on the basis of an exploration of the range of reasonable parameter values. The choice of method should be presented and justified.*

*Not relevant for our particular approach.*

**       Please discuss the scientific rationale for the choice of model structure and identify points where this choice could influence conclusions drawn. Please also describe the strength of the scientific basis underlying the key model assumptions.*

*Not relevant for our particular approach.*
