# Supplementary material for: Estimating population immunity against serotype-two poliomyelitis from the inactivated polio vaccine in routine immunization across 112 countries: A modelling study
Source: PLoS Med. 2026 Mar 19;23(3):e1004952. doi: 10.1371/journal.pmed.1004952 (PMC13001910; doi:10.1371/journal.pmed.1004952)
Supplement: Attachment — Submitted filename: Rev_resp_3.docx [file pmed.1004952.s006.docx]

GENERAL EDITORIAL REQUESTS
* Thank you for providing an Author Summary;  Ideally each sub-heading should contain 2-3 single sentence, concise bullet points containing the most salient points from your study. In the final bullet point of ‘What Do These Findings Mean?’ Please include the main limitations of the study in non-technical language. Please see our author guidelines for more information: [https://journals.plos.org/plosmedicine/s/revising-your-manuscript#loc-author-summary."](https://journals.plos.org/plosmedicine/s/revising-your-manuscript#loc-author-summary.%22)

Thank you, we have now included the limitations.

* Please confirm that your title complies with to PLOS Medicine's style. Your title must be nondeclarative and not a question. It should begin with main concept if possible. "Effect of" should be used only if causality can be inferred, i.e., for an RCT. Please place the study design ("A randomized controlled trial," "A retrospective study," "A modelling study," etc.) in the subtitle (ie, after a colon).

Yes.

* Please confirm that your abstract complies with our requirements, including format (three sections: Background, Methods and Findings, and Conclusions) and providing all the information relevant to this study type <https://journals.plos.org/plosmedicine/s/submission-guidelines#loc-abstract>

Yes

* Please confirm that all numbers presented in the abstract are present and identical to numbers presented in the main manuscript text.

Yes

GENERAL

* In the last sentence of the Abstract Methods and Findings section, please describe the main limitation(s) of the study's methodology.

We have now included these.

* In the author summary, in the final bullet point of 'What Do These Findings Mean?', please include the main limitations of the study in non-technical language.

This has now been included.

* Currently the majority of the Methods are in the supplements. Please provide more details (if not all)  on the Methods in the main text for transparency. As a reminder, we don’t have a word-limit.

We have expanded the methods section in the main text as follows:

*For each point in time between January 2024 and January 2031, we estimate for each country or territory the proportion of children under the age of five years immune to type-2 poliovirus by calculating, for each birthdate in the preceding five years the probability that a child has received one or two doses of IPV based on age eligibility, historic schedules and coverage at scheduled ages, before multiplying this by the seroconversion rate for the relevant age of administration. This calculation integrates any changes to immunization schedules or coverage during that window, with coverage levels held constant at 2023 values thereafter. These daily probabilities are then averaged, assuming a uniform age distribution and birth rate to give the overall immunity proportion. Population immunity reaches equilibrium five years after any new dose introduction or schedule change.*

FUNDING STATEMENT

* The funding statement should include: specific grant numbers, initials of authors who received each award, URLs to sponsors’ websites. Also, please state whether any sponsors or funders (other than the named authors) played any role in study design, data collection and analysis, the decision to publish, or preparation of the manuscript. If they had no role in the research, include this sentence: “The funders had no role in study design, data collection and analysis, decision to publish, or preparation of the manuscript.”

I believe I’ve done this correctly.

DATA AVAILABILITY

* Thank you for stating that the data is freely available, as well as providing the Github URL.

* Because Github depositions can be readily changed or deleted, we encourage you to make a permanent DOI'd copy (e.g. in Zenodo) and provide the URL.

This will be completed before publication

* Please include all-data sources in your data availability statement.

* Please include the statement on code availability in the data availability statement.

These will be included

* Regarding the seroconversion study data, please provide these data in a more easily accessible format. Are these data provided in Table 24 in the supplementary materials? If so, please specify this explicitly in the Data Availability Statement.  If not, please provide a table with all the data used in this study, as well as the sources these data came from.

The data table detailing the seroconversion studies is now included in .csv format in the linked repository

FIGURES
* Please define all elements of box plots in the figure caption - center line, box limits and whiskers.

This has now been completed.

* Please convert any stacked bar charts to another data representation for example a table, or other type of graph.

The stacked histogram has now been changed to single colour.

* Please consider avoiding the use of red and green in order to make your figure more accessible

The bars in figure 5 have now had their colours changed.

* Please confirm that the appropriate usage rights apply to the use of this map. Please see our guidelines for map images: <https://journals.plos.org/plosmedicine/s/figures#loc-maps>

Figure 1: please label the subpanels, so it’s clear for everyone which subfigure is referred to.

This has now been done, thanks.

#

1) Please provide a/amend your detailed Financial Disclosure statement. This is published with the article. It must therefore be completed in full sentences and contain the exact wording you wish to be published.

   1. State what role the funders took in the study. If the funders had no role in your study, please state: “The funders had no role in study design, data collection and analysis, decision to publish, or preparation of the manuscript.”
   2. If any authors received a salary from any of your funders, please state which authors and which funders.

These have all been included in the Acknowledgements section of the manuscript.

2) We have noticed that you have uploaded Supporting Information files, but you have not included a list of legends. Please add a full list of legends for your Supporting Information files after the references list.

This has already been included as requested in the Supporting Information information files after the reference list.

Comments from Reviewers:

Reviewer #1: Thank you for addressing my comment. I think my last point on confounding has not come through well enough - I do not doubt the data source to be very good. What I was not certain is the projected immunity from the national data. More details in the limitations around this, i.e. the projection inaccuracy, would be welcomed.

The text in lines 297-301 we discuss the limitations around the projection inaccuracy in terms of using national data.
